# The Feasibility of Equine Field-Based Postural Sway Analysis Using a Single Inertial Sensor

**DOI:** 10.3390/s21041286

**Published:** 2021-02-11

**Authors:** Sonja Egan, Pieter A. J. Brama, Cathy Goulding, David McKeown, Clodagh M. Kearney, Denise McGrath

**Affiliations:** 1 Institute for Sport and Health, School of Public Health, Physiotherapy and Sports Science, University College Dublin, Dublin D04 V1W8, Ireland; denise.mcgrath@ucd.ie; 2 Section Veterinary Clinical Sciences, School of Veterinary Medicine, University College Dublin, Dublin D04 V1W8, Ireland; pieter.brama@ucd.ie (P.A.J.B.); clodagh.kearney@ucd.ie (C.M.K.); 3 The Insight SFI Research Centre for Data Analytics, University College Dublin, Dublin D04 V1W8, Ireland; cathy.goulding@ucdconnect.ie; 4 School of Mechanical and Materials Engineering, University College Dublin, Dublin D04 V1W8, Ireland; david.mckeown@ucd.ie

**Keywords:** equine, postural sway, wearable technology, inertial sensor, postural control

## Abstract

(1) Background: Postural sway is frequently used to quantify human postural control, balance, injury, and neurological deficits. However, there is considerably less research investigating the value of the metric in horses. Much of the existing equine postural sway research uses force or pressure plates to examine the centre of pressure, inferring change at the centre of mass (COM). This study looks at the inverse, using an inertial measurement unit (IMU) on the withers to investigate change at the COM, exploring the potential of postural sway evaluation in the applied domain. (2) Methods: The lipopolysaccharide model was used to induce transient bilateral lameness in seven equines. Horses were monitored intermittently by a withers fixed IMU over seven days. (3) Results: There was a significant effect of time on total protein, carpal circumference, and white blood cell count in the horses, indicating the presence of, and recovery from, inflammation. There was a greater amplitude of displacement in the craniocaudal (CC) versus the mediolateral (ML) direction. A significant difference was observed in the amplitude of displacement in the ML direction between 4–12 h and 168 h. (4) Conclusions: The significant reduction in ML displacement during the acute inflammation period alongside greater overall CC displacement may be a compensatory behaviour for bilateral lameness.

## 1. Introduction

Postural sway (PS) has been described as the subtle sway during quiet stance [1], which facilitates the body’s ability to balance in any posture or activity [2]. PS is thought to provide information regarding postural control, i.e., the ability to maintain the body’s centre of mass (COM) within the base of support using somatosensory, vestibular, and visual sensory networks [3,4,5]. Perturbations such as visual occlusion have been reported to increase sway by up to 50% [6] while various standing tasks are used to assess balance deficits [7]. The COM is calculated as the summed average of the COM of all body segments [8] and can be captured via motion analysis or sensor technology. Postural sway refers to changes in the centre of gravity [9], often estimated through changes in the centre of pressure (COP), i.e., the weighted average of the pressures distributed over the surface in contact with the ground [8,10], measured using a force plate [11]. The COP is often referred to as the controlling variable and the COM the controlled. COP-COM is the scalar distance between the two variables at any given time, which is proportional to the horizontal acceleration of COM [12]. The relationship between these three variables is based on the inverted pendulum theory [12]. Postural sway captured during periods of quiet standing is a quantifiable movement metric that has been used extensively to classify health, aging, and disease-related changes in humans for over three decades [1,5,13,14].

Sway is thought to be related to inherent delays or error within the sensory-motor feedback control system [15,16], suggested to be due to the COP reacting to the estimated position of the COM displaced through events such as breathing and general muscle activity. There is a strong negative relationship between the COP and the COM, which suggests that when the COP is ahead of the COM the direction of acceleration is backward and vice versa [11]. However, over an extended period of time the average of the COP must equal the average of the centre of gravity in order to maintain balance [11]. Carpenter et al. (2010) has suggested that sway may also be a form of exploratory behaviour used by the central nervous system to adapt to internal and external changes [15].

The concept of equine postural control has been investigated in recent years [17,18,19,20,21]. Most of these studies have been undertaken in a laboratory setting using force or pressure plates to measure COP magnitude and velocity in craniocaudal and mediolateral directions. Equine studies have indicated, in line with human research, that quantification of postural sway may be useful to detect neurological problems such as ataxia and musculoskeletal issues resulting in gait abnormalities [17,18,22,23]. Postural sway thus appears to be a promising metric to characterise health states in equines. However, the impracticality of availing of a bespoke movement laboratory limits its use in the applied field. On-body inertial sensors have been used to quantify postural sway in humans [24,25,26] and to a lesser degree in equines. Moorman et al. (2016) was the first to compare the use of a withers mounted portable media device to a force plate, investigating equine postural stability [27]. Advances in technology opens the possibility of capturing, unsupervised postural sway data using wearable or ambient sensing, as a means of assessing the health status of a horse in the applied setting. Given that completely quiet standing in equines would be considered more the exception rather than the rule, it is necessary to characterise the incidence of this behaviour, if postural sway is to be pursued as a feasible metric in both field-based experimental work and for future monitoring approaches in the applied setting.

Bilateral lameness is a complex condition which is difficult to detect due to the apparent symmetry present between left and right sides [28,29]. Both subjective and objective methods of lameness evaluation utilise asymmetry among other features to determine the presence and severity of unilateral lameness [30]. There is comparatively less research investigating the expression of bilateral lameness in equine movement behaviours. Postural sway could potentially be a useful indicator of bilateral inflammation as it may capture patterns of subtle weight shifts in limb pairs. If such a change could be identified in horses in a real-world monitoring scenario, this would enable early identification and intervention for bilateral conditions that are otherwise difficult to detect.

The purpose of this study was to explore the feasibility of capturing useful postural sway data in an applied setting using a single withers fixed sensor. An induced bilateral lameness model was applied in the forelimbs and evaluated over seven days to understand if the postural sway signal in quiet standing was useful in detecting inflammation and recovery. The estimated equine COM position in square stance is near the dorsal process of the 13th thoracic vertebrae [31] and is not directly measurable [9,12]. The rationale for the wither’s placement was to capture posture sway at a globally stable point that could pick up changes due to bilateral lameness. This rationale is supported by the commonly used lumbar sensor position in human research, preferred due to its proximity to the COM and lower limbs [4,10]. This study was guided by two main aims. The first aim was to use concurrent video annotation and an inertial sensor to determine the frequency of completely quiet standing in equines. It is important to determine the frequency of quiet standing, and to understand how tolerant we can be of minor movements during these stationary periods, if the data is to be used as a meaningful metric of health. The second aim sought to explore the impact of induced bilateral lameness on equine postural sway as it is not known if horses sway more or less, or with more variability in bilaterally inflamed conditions, or how this changes over a period of recovery. A recent meta-analysis on human postural sway research has highlighted the problem of assuming a one-sided change in movement variability (i.e., that it is always increased or always decreased due to disease) through pooling of data into a single data set that is then averaged, potentially masking individual trends [32]. Authors suggested that the position of each case within the population should be examined. This is especially true for equine postural sway given that this is a relatively unexplored area where the direction of change in postural sway variability is not known, and thus this is the approach taken in this study.

## 2. Materials and Methods

This study was approved by the University College Dublin, Animal Research Ethics Committee (AREC-16-29-Brama) and the Health Products Regulation Authority (AE18982/P105) in compliance with Irish legislation on animal experiments. Seven horses (5 mares, 2 geldings), age (14.5 ± 2.4 years), weight (366.5 ± 26.3 kg), were included. Subjects were stabled individually in single boxes (4 × 4 m) on wood shavings with daily concentrate, regular hay and water provided ad libitum. Horses were settled into the stables 14 days prior to data collection to facilitate familiarity with their routine and environment. Each stable had three walls and a front grid bar design allowing the animals to look out the front of their stable and place their head over the door.

### 2.1. Lameness Induction

The left and right dorsal carpal regions of each equine were clipped and prepped for dorsal arthrocentesis. Transient induced bilateral lameness was induced using lipopolysaccharide from Escherichia coli O55:B5 (catalogue number L5418; Sigma-Aldrich Ireland Ltd., Arklow, Co. Wicklow, Ireland) was diluted to a final concentration of 0.25 ng/mL in sterile lactated Ringer’s solution. Animals were sedated, if necessary, with xylazine (0.2–0.5 mg/kg intravenously, Chanazine 10%^®^; Chanelle, Ireland) and butorphanol (0.02–0.04 mg/kg intravenously; Alvegesic vet 10^®^, ALVETRA u. WERFFT GmbH, Boltzmanngasse 11, A-1090 Vienna, Austria). Arthrocentesis was performed with a 20 G × 40 mm needle and 1 mL LPS solution was delivered aseptically into both left and right intercarpal joints after withdrawal of a synovial fluid sample.

### 2.2. Data Collection

Horses were monitored intermittently using both video footage and IMU technology on non-consecutive days until 168 h post lameness induction. The lameness induction protocol was repeated 3 times over a period of 8 weeks. This included three 7–14-day wash-out periods during which horses were put out to pasture to recover. Data monitoring took place continuously for 12 h on day 1 (expressed in two-hourly periods: 0, 2, 4, 6, 8, 10, 12 h) and approximately 6–8 h at 24 h, 72 h, and 168 h post induction.

**Figure 1:**
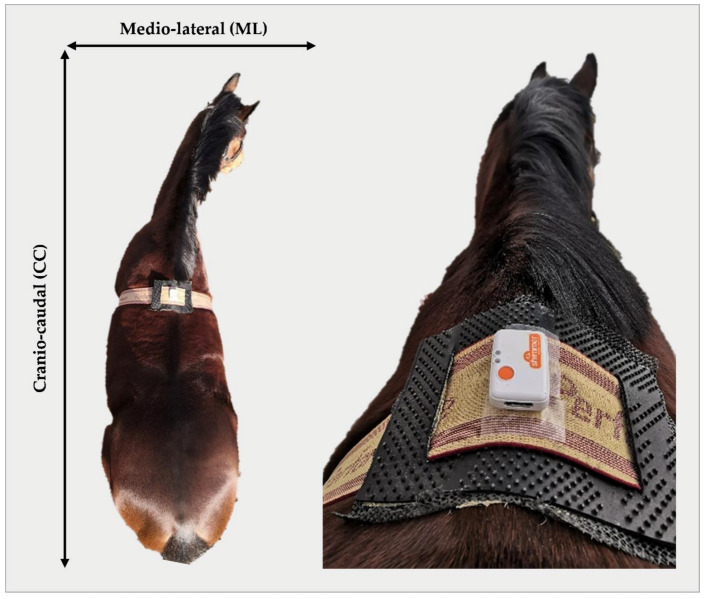
IMU withers placement. A bird’s-eye view of the IMU placement on the withers via a standard surcingle with the bespoke non-slip attachment. This image is for demonstration purposes to facilitate a view of the IMU on the horse. The IMU attachment was reinforced with additional adhesive during the experiment.

Each horse was equipped with an IMU (SHIMMER, Shimmer Research, Dublin, Ireland), placed on the withers. The sensor was fitted using a custom-built attachment via a standard surcingle (Figure 1.). The IMU was configured to stream tri-axial low noise accelerometer (±2 g), wide range accelerometer (±16 g), gyroscope (±2000 °/s) and magnometer (±1.3 Ga) data. Data were collected at a sampling rate of 102.4 Hz using the SD log firmware and configuration settings which were set in the ConsensysPRO^TM^ (v1.0.0) software (SHIMMER, Shimmer Research, Dublin, Ireland). Similarly, data were imported and downloaded for offline analysis using the ConsensysPRO^TM^ application.

A single Hikvision 4 Megapixel EXIR IP PoE turret Camera with 4 mm lens was fixed to the upper right side of each stable, wired out of reach of the animal. This allowed for continuous recording without interfering with normal equine behaviour. Cameras recorded footage throughout the above specified time points to a 4 terabyte Skyhawk CCTV hard drive for download, offline analysis, and video annotation. Videos were sampled at 20 frames/s with a 1280 × 720 resolution.

Synovial fluid samples for biomarker analyses were obtained at timepoints: 0, 8, 24, 72, and 168 h. These were taken by the same boarded equine specialist (diplomates European College of Veterinary Surgeons (ECVS) who has extensive experience in the procedure (C.M.K). Part of the synovial fluid aspirate was placed in EDTA tubes for white blood cell count (WBCC) determination by haemocytometry and total protein (TP) measurement using a refractometer, as objective indicators of joint inflammation occurring in the model over the timeline of the experiment. Carpal circumference was measured at the level of the accessory carpal bone with a tape measure. Similarly, this measurement was taken by the same equine boarded specialist (C.M.K.). The skin at this point was marked with ink to ensure consistency in measuring technique, the smallest unit of measurement on the tape was 1 mm.

### 2.3. Data Processing

Seven horses were included in the analysis of the frequency of quiet standing periods and markers of inflammation. To explore the individual recovery profile of each horse with respect to postural sway, for a horse’s data set to be included in the analysis, an inclusion criterion of at least seven out of 10 timepoints across a 1-week experimental period containing a 60-s quiet standing period was established. This led to the exclusion of Horse 4 from further analysis. The data set with the most data points was extracted for each of the 6 remaining horses to explore the changes in postural sway following induced bilateral lameness and subsequent recovery.

#### 2.3.1. Quiet Standing: Video Annotation

Video annotation was undertaken as per a protocol previously published by the authors [33]. In brief, equine behaviours of interest were defined by the research team and an annotation framework was developed. Two veterinary students (experienced in the annotation of animal behaviours) were trained in the annotation framework and they assisted with its initial piloting. Following successful reliability testing of the annotation system, both annotators manually annotated 24 h of the CCTV footage of equine stable behaviours together before proceeding to annotate separately. A third annotator (S.E) spot checked approximately 40% of all the annotated data against the CCTV to ensure reliability of annotation system. Quiet standing CCTV footage was annotated under the following definition: the horse had all four hooves in contact with the ground or resting a hindlimb—weight shifts are included; not intently interested, head drooping, gentle ear flicker or looking or listening with intent/interest allowed, ears fully pricked, cannot be leaning on another surface or progress away from original position, quiet standing activity was not captured if the horses’ head was over the door. Horses were not held or intentionally distracted by handlers and hoof/limb positions were not standardised.

#### 2.3.2. Quiet Standing: IMU Processing

**Figure 2:**
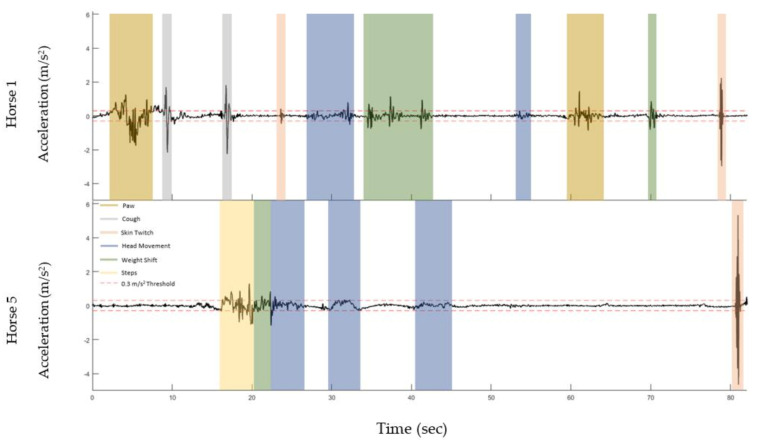
A sample of labelled acceleration signal examples of quiet standing and other activity. Examples of two sections of acceleration signals extracted from two different horses illustrating the varying magnitudes of different stationary and locomotor activities. Dark yellow: Paw, Grey: Cough, Orange: Skin twitch, Blue: Head movement, Green: Weight shift, Pale yellow: stepping and dotted red line: 0.3 m/s^2^ threshold.

Acceleration data were sampled at 102.4 Hz and bandpass filtered between 0.1 and 10 Hz. The data were transformed into a global coordinate system using the gradient descent algorithm described by Madgwick et al. [34]. The acceleration signal for each horse was inspected alongside their own video footage to determine an appropriate acceleration threshold that represents quiet standing (i.e., no extraneous movement). Quiet standing activity was extracted from the original video annotation excel file for trials 1–3, combined and organised into two-hourly time points (i.e., 0 h, 2 h, 4 h, etc.). A "quiet" threshold of 0.3 m/s^2^ in the ML direction was chosen based on this manual pairing and inspection of video and sensor signals across horses. This threshold also aligned to previous equine postural sway work presented by Moorman et al. using a portable media device at the withers to capture quiet standing [27]. The data presented in Moorman et al. demonstrated that the mean acceleration ranges in both ML and CC directions (Figure 1) were between 0.28 and 0.32 m/s^2^. The threshold was rounded to 0.3 m/s^2^ for this purpose of this research. The acceleration signals derived from the six horses included in the dataset were analysed to determine the suitability of this threshold to these data. Based on the behaviours evident in Figure 2, and previous research, the 0.3 m/s^2^ threshold was judged to be the maximum appropriate threshold value and was applied for further data segmentation and analysis. Quiet periods lasting 65 s were then automatically extracted based on this threshold. The initial and final 2.5 s were removed, and the remaining 60 s were used for further analysis.

Outcome measures from existing postural sway research were derived from the acceleration signal: Cranio-caudal (CC) and medio-lateral (ML) total displacement, amplitude of displacement and path length. All horses had multiple instances of quiet standing during the final 168 h testing period across trials 1, 2, and 3. An assumption was made, based on the outcomes of biomarker analyses, that the horses had recovered by 168 h. Additionally there were less environmental distractions on day 7 versus day 1. Thus, postural sway values in the 168 h timepoint were averaged across trials 1, 2, and 3 to create a "postural sway" baseline for each horse.

### 2.4. Displacement Analysis

ML and CC displacement were obtained by twice integrating the horizontal acceleration signal. Low frequency drift was reduced using a second-order polynomial fit. The signal was high pass filtered at 0.1 Hz and the mean amplitude of the signal was subtracted before and after each integration procedure. The peak-to-peak amplitude of displacement in each direction was then calculated.

### 2.5. Path Length

The total path length was obtained by summing the Euclidean distance between consecutive pairs of displacement data points in the ML and CC directions [23].

### 2.6. Statistical Analysis

Repeated measures ANOVA were completed on the averaged (trial 1, 2 & 3) inflammation markers (white blood cell count, carpal circumference, and total proteins) to determine the effect of time on changes in synovial fluid and joint inflammation. The Huynh–Feldt correction was applied where sphericity was violated, and the Bonferroni adjustment was used to account for multiple comparisons.

Dependent t-tests were undertaken to compare PS metrics during the acute and recovered period. We defined the acute period as 4–12 h post-lameness induction based on the kinematic gait analysis of the horses in this experiment (under review) and in line with previous LPS research [35,36]. The alpha value was set at 0.05.

## 3. Results

### 3.1. Frequency of Quiet Standing

**Figure 3:**
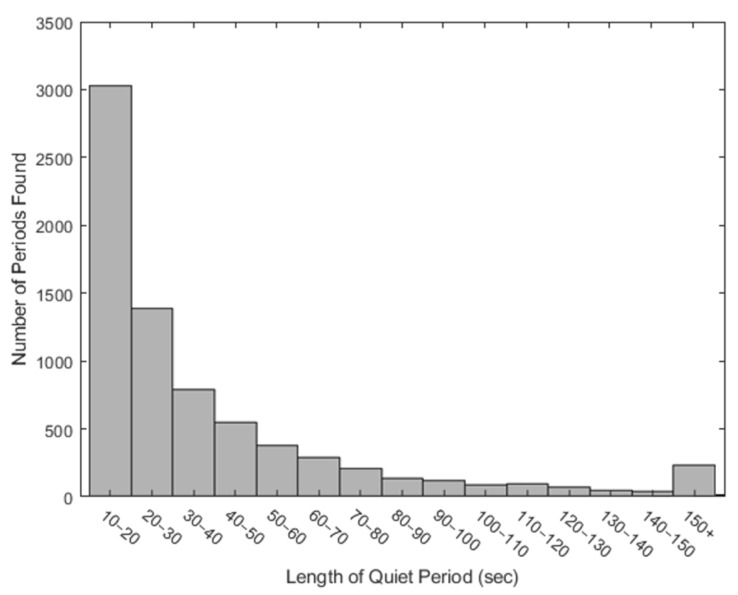
Total number of quiet standing periods. The number of quiet standing periods extracted through the application of 0.3 m/s^2^ acceleration threshold across all horses and trials, verified by video annotation.

**Figure 4:**
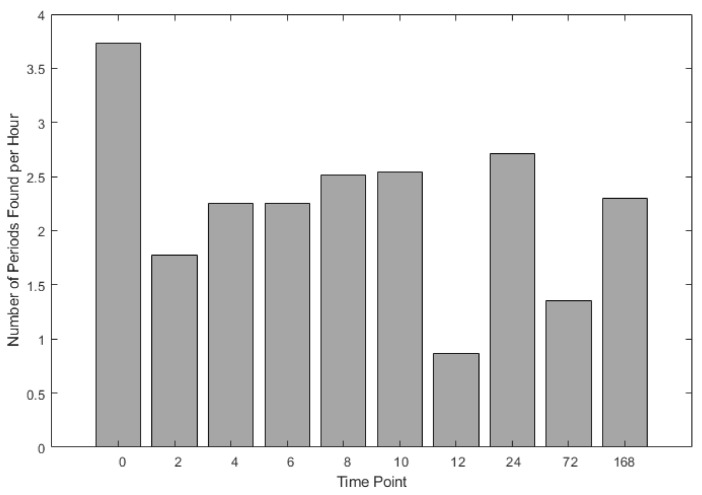
Number of quiet standing periods per hour. The number of 60 s quiet standing periods per hour across all horses and trials.

Application of the 0.3 m/s^2^ thresholds resulted in the extraction of approximately 500 quiet standing samples lasting 60 s or more across the experimental period (Figure 3). Across horses, 60 s of quiet standing activity occurred on average twice per hour (Figure 4) and accounted for 2–12% of behaviours in each hour period.

Comparison of several acceleration signals during quiet standing with simultaneous video footage of the horses demonstrated that it is not feasible to classify the different movements that occur during stationary periods according to acceleration ranges. This is because acceleration ranges for movements such as skin twitching, subtle head movement or pawing are not consistent within or across horses, illustrated in Figure 2. For example, the magnitude of the acceleration signal registered during a skin twitch changes depending on where it occurs.

### 3.2. Inflammation Markers

**Figure 5:**
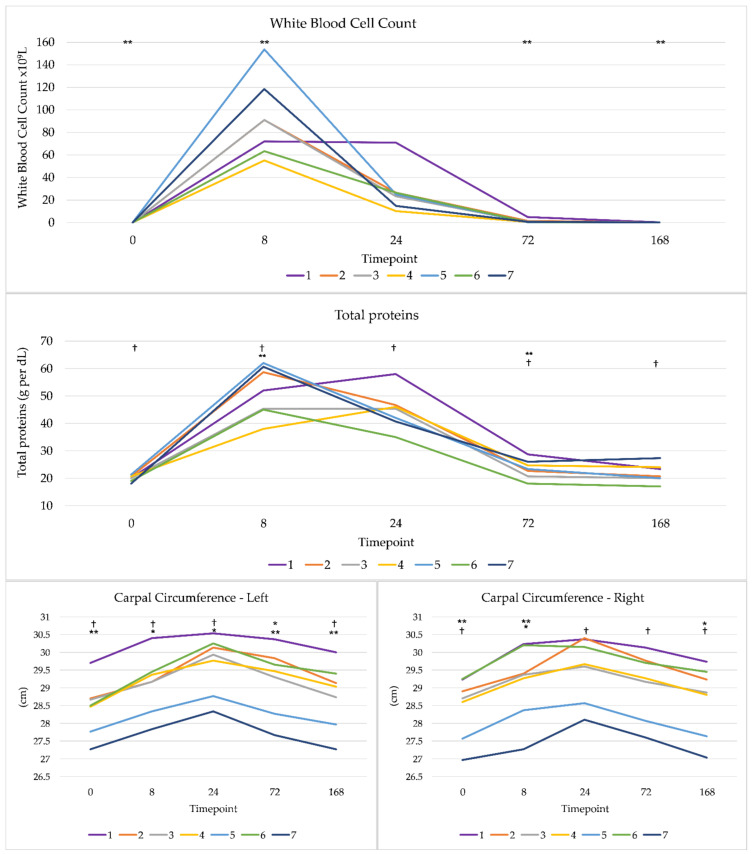
Inflammation markers. White blood cell count, total proteins, and carpal circumference across the 7 horses, * denotes *p* ≤ 0.05, ** denotes *p* ≤ 0.005 and † *p* ≤ 0.001. See Section 3.2 for list of significances across timepoints.

All joint inflammation markers displayed a significant effect of time over at least three timepoints. Repeated measures ANOVA identified a significant main effect of time for change in Carpal Circumference in the left and right forelimbs (*p* = 0.000). Left limb pairwise comparisons revealed significances between the following timepoints: 0 h and 8 h (*p* = 0.001), 0 h and 24 h (*p* = 0.000), 0 h and 72 h (*p* = 0.005), 8 h and 24 h (*p* = 0.019), 24 h and 72 h (*p* = 0.009), 24 h and 168 h (*p* = 0.000) and 72 h and 168 h (*p* = 0.003). Right limb pairwise comparisons revealed significant differences between the following timepoints: 0 h and 8 h (*p* = 0.003), 0 h and 24 h (*p* = 0.000), 0 h and 72 h (*p* = 0.001), 8 h and 168 h (*p* = 0.013), 24 h and 72 h (*p* = 0.001), 24 h and 168 h (*p* = 0.000) and 72 h and 168 h (*p* = 0.001). A second repeated measures ANOVA identified a significant main effect of time on white blood cell count (*p* = 0.000). Pairwise comparisons identified significances between: 0 h and 8 h (*p* = 0.004), 8 h and 72 h (*p* = 0.005), and 8 h and 168 h (*p* = 0.004). A third repeated measures ANOVA identified a significant (*p*= 0.000) main effect time on total protein count. Pairwise companions were significant between: 0 h and 8 h (*p* = 0.001), 0 h and 24 h (*p* = 0.001), 8 h and 72 h (*p* = 0.002), 8 h and 168 h (*p* = 0.001), 24 h and 72 h (*p* = 0.000) and 24 h and 168 h (*p* = 0.001). We therefore conclude that the horses were experiencing a degree joint inflammation and associated discomfort up to 24, and in some cases 72 h (Figure 5).

### 3.3. Displacement Analysis

CC amplitude of displacement was always greater than the amplitude of displacement in the ML direction (Table 1). Comparison of CC and ML amplitude of displacement at the 4–12 h period versus 168 h period (Table 2) identified a highly significant difference between the 4–12 h and 168 h timepoints for ML amplitude (*p* = 0.005). There was no significant difference between timepoints for CC amplitude of displacement (*p* = 0.15). There were no statistically significant differences in CC or ML total displacement were found (*p* = 0.42; *p* = 0.51, respectively).

### 3.4. Path Length

**Figure 6:**
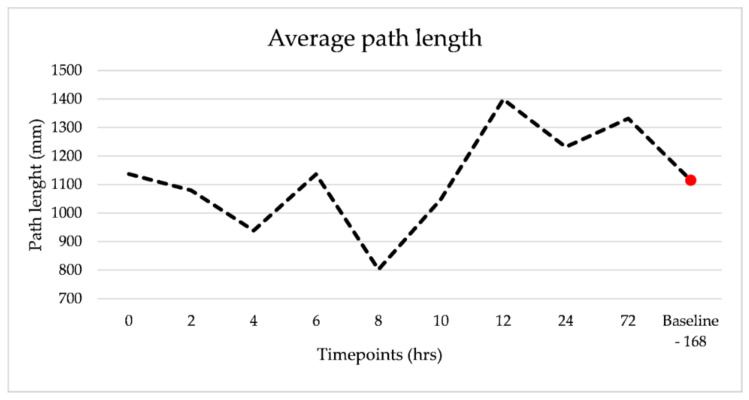
Average change in path length. The average change in path length for the six horses over all captured timepoints. The red circle indicates the “baseline” value as described in the methods section.

Values for path length were condensed into the acute 4–12 h (1044.8 mm ± 199.3 mm) window and compared to the 168 h (1115 mm ± 229.5 mm) baseline (as described in the methods section). T-Testing identified no significant difference between the two timepoints (*p* = 0.61). Inspection of the average path length trend line (Figure 6) across the six horses demonstrate a reduction in path length between 2 and 10 h post bilateral lameness induction. Path length increased again at 12 h gradually reducing toward the 168-baseline value. There was no statistically significant difference between timepoints (*p* = 0.61).

### 3.5. Stabilograms

**Figure 7:**
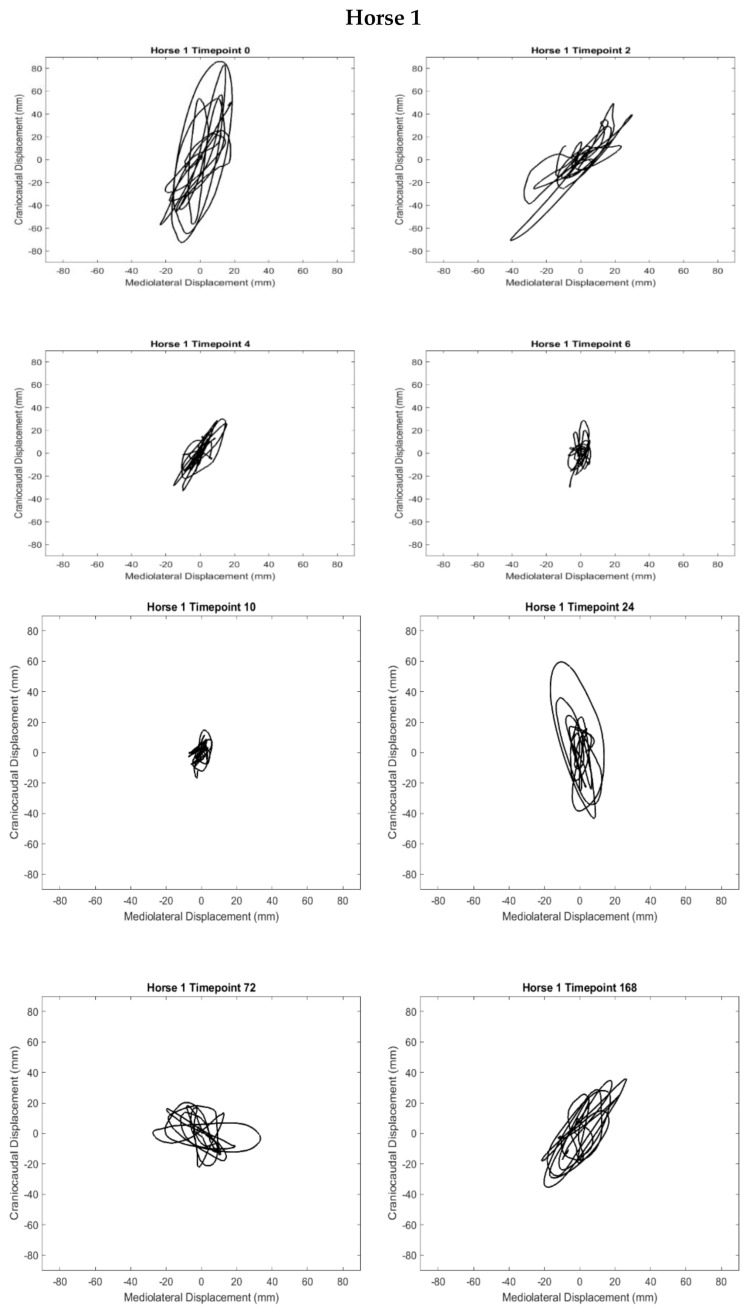

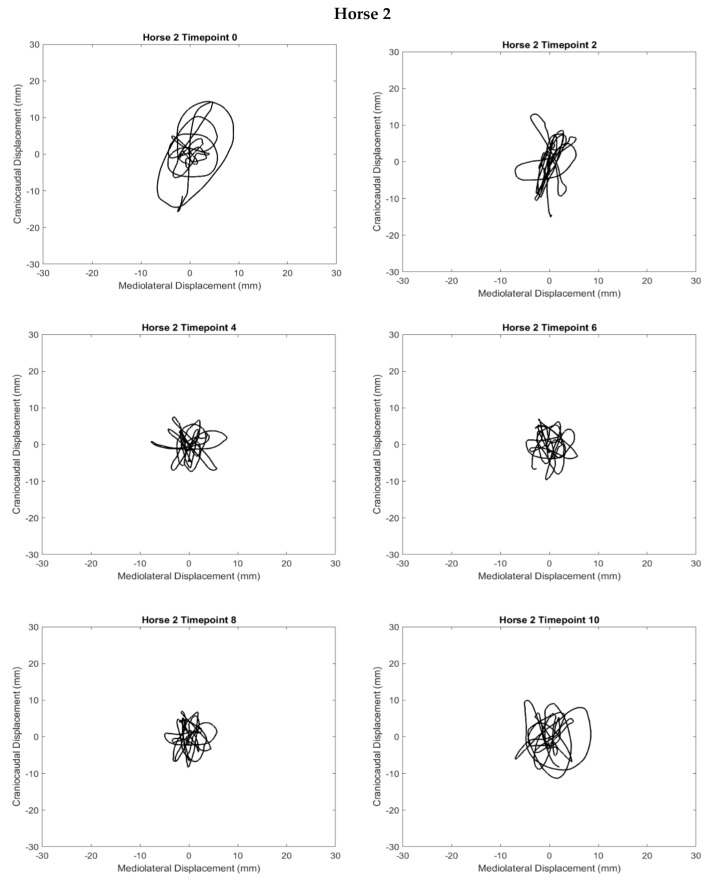

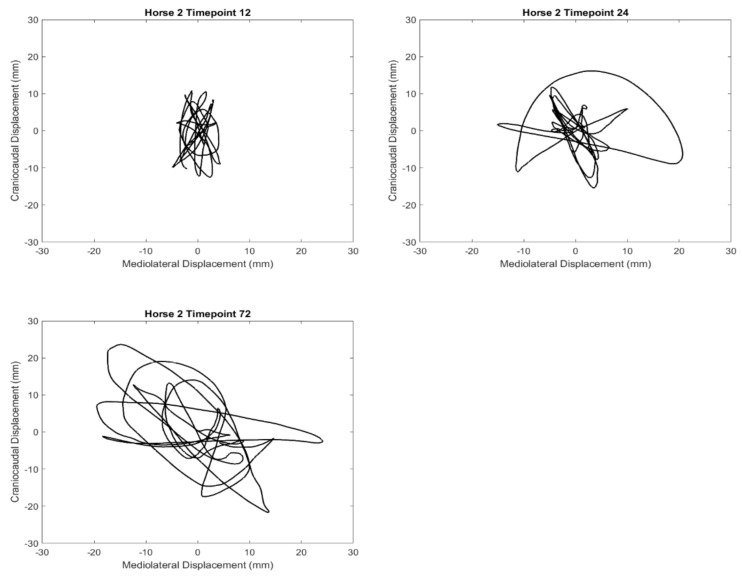

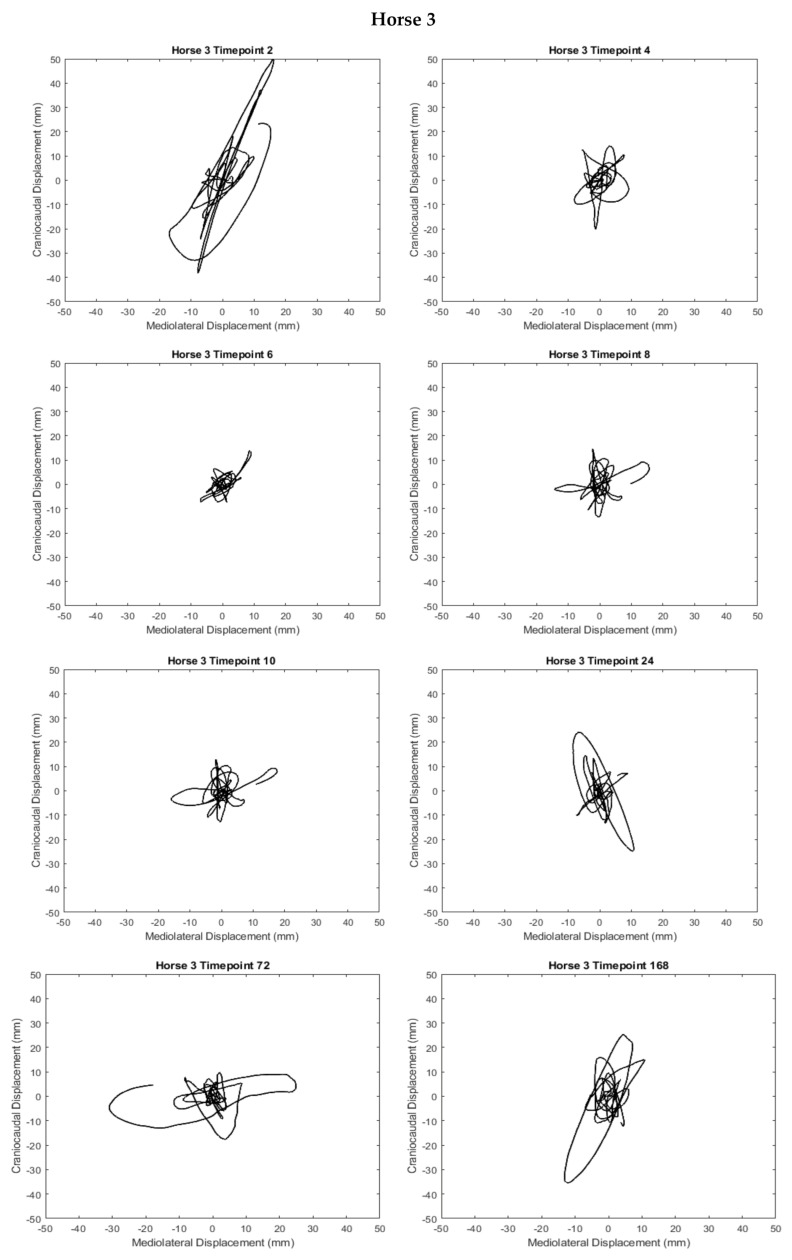
Stabilograms for horses 1, 2, and 3.

Postural sway at the withers was visualised using stabilograms that show horizontal sway in CC and ML directions over a 60 s period. Stabilograms were produced for each horse for all available timepoints. Stabilograms for horses 1, 2, and 3 (Figure 7) are shown here with data for other subjects plotted in Supplementary File 1. Visual inspection of these stabilograms suggest that the naturally occurring variations in postural sway were constrained during the most inflamed period for all but one horse (H6) in this study.

## 4. Discussion

The force plate is considered the gold standard measure for human balance, equine kinetics, and equine COP measurement [7,10,17,20,22], but it is not transferrable to an applied setting. Pressure mats have been shown to be a potential solution [23]; however, the portability and cost of inertial sensors compared with force and pressure plates [5] enables the potential of unsupervised postural sway monitoring in the equine domain. A 2020 systematic review of wearable technology to assess peripheral neuropathy identified the feasibility, accuracy, and validity of wearable devices in discriminating between healthy and balance impaired human populations [4]. Najafi et al. 2010 reported a strong correlation (r = 0.92) for balance features derived from pressure platforms and wearable technology in all study conditions [4,37]. This research sought to expand the understanding of the incidence of naturally occurring quiet standing in equines and the feasibility of using wearable sensor technology to detect bilateral lameness-related changes in equine postural sway. There was a significant effect of time on change in carpal circumference, white blood cell count, and total protein resulting from induced bilateral lameness, indicating that there was carpal joint inflammation present at the acute stage (i.e., day 1 of the study) that returned to normal levels by the end of the study. It was identified that 60 s of quiet standing occurs approximately twice per hour across horses. The data showed that the frequency of quiet standing periods was not drastically affected by induced bilateral lameness, however certain characteristics of the postural sway appears to have been affected during the acute inflammatory period. There was a statistically significant reduction in ML amplitude of displacement in the 4–12 h period versus the final 168 h period—when the horses were assumed to have returned to normal, indicating that the horses’ sway in quiet standing was reduced when bilaterally inflamed. The stabilograms derived from these data suggest that the level of variability in the sway micro-movements can be meaningfully tracked over a period of inflammation and recovery using intuitive visual aids. From a human–computer interaction standpoint, this indicates that postural sway could be an attractive metric that could potentially be monitored by non-expert stakeholders in an applied setting. This is the first study to show the feasibility of longitudinal postural sway measurement in the applied field.

Bilateral lameness status was confirmed between 4 and 12 h through biomarkers of inflammation and two boarded equine specialists (diplomates European College of Veterinary Surgeons (ECVS)). Previous research has shown a reduction in locomotor activity and increased stationary behaviours in equines in painful states [38], which was not born out across the 3 × 1 week experimental periods examined in this study. The subtle lameness induced, coupled with the stringent threshold applied to the acceleration signal that led to the elimination of most of the extraneous movement, could explain this discrepancy. It appears that characterising specific types of movements based on acceleration thresholds or ranges across horses with a view to including or excluding those movements from the analysis is not a feasible approach. For example, head and neck movements that occurred while stationary registered acceleration values that were often lower than the panniculus reflex or skin twitch that occurred at the site of the sensor (Figure 2). Thus, moving forward with this research, we would only consider using the lower threshold of 0.3 m/s^2^ to remove almost all extraneous movement, leaving only quiet standing.

Research in human subjects has demonstrated that trial lengths of at least 60 s [39,40] are required to create a representative picture of the sensorimotor system as they limit erroneous findings related to the variability of a non-stationary signal. Laboratory equine postural sway trials are often discounted or discontinued on visual recognition of motion [19,27,41], which inhibits the widespread application of sway analysis. Further to this, trial lengths in previous equine research have ranged between 8 and 60 s during which horses are loosely restrained to discourage movement. Horses often must be trained to stand quietly for long periods, which Clayton and Bialski have highlighted is not practical prior to clinical assessment in quiet standing [17,20]. Our findings demonstrate that capturing a minute of completely quiet standing, unsupervised using a wearable inertial sensor is feasible in both healthy and inflamed states.

Previous equine research has suggested that COP amplitude of displacement is greater in the direction of the smaller base of support [22,23,41] i.e., the ML direction in horses, which can be further exacerbated by narrow hoof stance. This is suggested to be related to the limbs’ anatomical structure—designed for greater movement in the sagittal plane and overall larger base of support in the CC direction, enabling greater stability [23]. However, relatively small absolute values and differences in the amplitude of COP displacement between ML and CC directions (~4 mm) have been reported [18,22], indicating a high level of overall stability in both directions in the quadruped compared with humans. This is to be expected given the much larger base of support. Indeed, absolute COP amplitude in humans have been reported to be three time larger in the anterior–posterior direction and 50% larger in the ML direction than in horses [22,23]. In this study, greater amplitude of displacement in the CC versus ML direction was observed at baseline and during the inflamed period. Research from Clayton et al. similarly illustrated greater range of motion in the CC direction versus the ML direction, suggested to be due to subtle movement in the head and neck; which accounts for 10–14% of the horses’ total body mass [17].

It is difficult to compare our data to other equine research for a number of reasons. Trial lengths of 60 s are not common in equine research and are likely to capture a wider amplitude of sway and path length than in shorter trials. Foot position was deliberately not controlled in this study to see if postural sway parameters could provide meaningful information about health status, even in an unsupervised, real-world setting. No studies have previously investigated postural sway in bilateral lameness. Finally, most of the existing equine postural sway research has investigated equine postural sway based on changes in COP. Only one other study has used wearable technology to capture postural sway, and found that foot position did impact outcome measures [27]. Here we examined postural control by tracking sway at the withers, which is closely located to the body’s COM. It can be expected therefore that the absolute amplitude ranges would be different, due to the height of the sensor off the ground yet related in terms of patterns of movement according to the inverted pendulum model. The data suggests that the ground reaction forces exerted by the inflamed forelimbs are constraining the movement of the COM. Hence the stabilograms presented are a useful way to interpret the experimental outcomes.

The significant reduction of ML amplitude of sway in the 4–12 h period may be related to a change in the systems’ exploratory behaviour [15]. A recent meta-analysis by König et al. [32] demonstrated that in neurological conditions, human pathological subjects demonstrate higher variability in postural sway, as measured by sway area. However, there is an alternative hypothesis that suggests that there is a zone of “optimal variability” in a biological system that determines health status, where higher levels of variability may not necessarily be “bad” and lower levels may not necessarily be “good” [42]. The idea of “optimal variability” that has been discussed in the human movement literature [32,43] suggests that a healthy motor control system is variable, explorative and adaptive and an impaired system is either too rigidly constrained or uncontrolled. For example, sitting postural sway research in infants suggested that the development of postural control strategies involves the exploration of multiple degrees of freedom in order to develop an adaptive control strategy, suggesting that the variability manifested as postural sway also has a functional, exploratory role. The stabilograms provided here show that five out of six horses exhibited particularly constrained postural control that coincided with periods of acute inflammation. This is suggestive of a motor control system that has become rigid and protective. Thus, it appears that while bilateral inflammation does not impact the frequency of quiet standing behaviour, it does impact the variability of postural sway. Human limb pain has been suggested to alter postural stability due to altered proprioceptive input—a similar mechanism in horses is likely [44]. Further research into equine postural sway should be open to the possibility of a two-sided change in postural sway variability, with appropriate statistical analysis that can recognise non-linear trends in longer times series.

Path length investigates two-dimensional change in ML and CC directions, here presented as total path length. It is difficult to compare the changes in path length to existing equine research due to the longer duration of our quiet standing periods and lack of other IMU postural sway research. T-testing was applied to examine the difference in path length between 4–12 h and 168 h returned no statically significant differences. In humans, a longer total path length has been suggested to be due to postural instability [45]. However, Rhea et al. (2014) has suggest that such an interpretation of path length may determine that two individuals share the same total path length in a given time, but provide no additional information regarding how individual postural control strategies were applied [45]. Thus, it is important to look at additional factors, such as the stabilograms in Figure 7, to understand how postural control strategies differ between individuals and indeed over time. The stabilograms highlight the greater changes in ML excursions versus CC during the acute period. There is limited IMU-based research on equine PS, particularly in a non-laboratory-based setting. The parameters we have reported in this study mirror previous research in horses and focuses on the amplitude of movement and the amount of movement variability exhibited during inflamed and recovered periods. These parameters were chosen as first targets in this exploratory study as these were expected to show changes in bilateral lameness and are relatively robust (compared to other metrics of movement variability in PS such as sample entropy which is based on time series analysis [46]). Research in human movement has provided us with many more possible postural sway parameters that one could derive from this data which could provide useful insights into the changes in equine health status over time. However, in advance of further investigations into the dynamics of the motor control system due to real-world injury or fatigue using wearable sensing tools, further feasibility work is advised with respect to the uncontrolled environment where foot position is/is not standardized [47].

The authors have outlined the concept of a “smart stable” [21], where integrated technologies could be used to support equine health and management automatically. There are many wearable and ambient technologies now available that could be exploited to realise this automated vision. Placidi et al. [21] found that over 95% of postural sway data collected with a 3D video camera system was not significantly different to a traditional force plate method in humans. Wang et al. [48] demonstrated that inexpensive webcams can be used to calculate COM position in human standing and walking activities. There is no doubt that instrumentation of stables has the potential to support equine management and welfare in a much broader sense than has been realized to date. These technologies could be combined with sensor outputs to confirm what these movements "look" like from a stabilogram perspective and improve end-user interaction with quantitative data in the future. However, the question remains whether the field is ready and desires these developments [21].

## 5. Conclusions

This research demonstrates the basic capabilities of postural sway monitoring using a singular inertial sensor unit. This is the first study to investigate how often and for how long equines settle into a state of quiet standing which would enable the analysis of postural sway captured in the applied setting. Using a 0.3 m/s^2^ threshold on the acceleration data in the quiet standing periods, almost all of the extraneous movement was excluded. The statistically significant reduction in ML displacement during the acute inflammation period alongside greater overall CC displacement may be a compensation behaviour in bilateral lameness specifically, which has not been reported on previously. The reduction in ML sway during the acute inflammation period may be due to a reduction in exploratory behaviour. We submit that conclusions on the health of the sensorimotor system based on postural sway depends both on the context in which it is measured (e.g., motor development, neurological disease, and limb injury), the particular metric of postural sway that is analysed and whether or not it is in a laboratory or field-based environment. The next steps in field-based monitoring of postural sway using IMUs should seek to: understand more clearly the natural day-to-day biological variability of postural sway in equines, explore the possibility/need to standardise foot positioning over long monitoring periods, examine a wider range of additional PS outcome measures, and investigate sensor-video integration capabilities to enable unsupervised monitoring. Additionally, the onset and recovery of bilateral lameness versus unilateral lameness should be explored with respect to postural sway. This could potentially uncover further insights that could distinguish complex lameness and be applied in longitudinal monitoring paradigms to enable early intervention and recovery.

## Figures and Tables

**Table 1:** Amplitude of displacement data.

Cranio-Caudal Amplitude of Displacement (mm).										
	0	2	4	6	8	10	12	24	72	(B)168
**H1**	−47.1389	−11.9613	4.381976	20.52378		−12.3364		−1.07204	4.954598	8.725844
**H2**	106.2469	113.4973	66.96512	108.7198	101.6003	185.0028	194.7217	120.1628	148.056	
**H3**		147.4473	88.46477	66.86672	78.10156	54.81889		146.0614	99.52632	121.2145
**H5**	93.70621		69.37689	174.8362	45.59515			117.3788	107.8154	111.2028
**H6**	10.32397		10.36763			30.9781	−2.25987	8.367317	0.397835	4.997202
**H7**	100.9326	94.19014	166.9199	164.0935	106.6934	91.54561	165.7867	132.5918	112.0737	103.5486
**Average**	52.81416	85.79335	67.74605	107.008	82.99759	70.00182	119.4162	87.24835	78.80398	69.9378
**SD**	61.05513	59.57178	54.12221	58.22919	24.13744	66.66681	86.84508	59.90119	55.93903	51.81886
**Medio-Lateral Amplitude of Displacement (mm)**										
	**0**	**2**	**4**	**6**	**8**	**10**	**12**	**24**	**72**	**(B)168**
**H1**	16.34772	−8.55322	−4.36109	−5.72143		10.4833		5.174975	9.216272	−3.00699
**H2**	78.52314	91.96439	35.15835	72.05765	69.33291	82.08802	69.93044	77.17923	80.33109	
**H3**		55.80658	56.02084	38.62816	51.44049	60.04469		53.10575	40.92368	41.90341
**H5**	57.27578		48.12936	74.4663	56.4325			106.4024	75.84028	76.68095
**H6**	−11.7363		1.914441			33.31275	9.042947	−9.63795	−5.04264	1.037078
**H7**	63.91576	71.36391	98.04188	67.3006	74.33996	52.4736	74.58922	96.74821	80.19373	72.48259
**Average**	40.86522	52.64542	39.15063	49.34626	62.88647	47.68047	51.18753	54.82877	46.9104	37.81941
**SD**	33.42689	37.58866	34.47014	30.38589	9.292512	24.28905	29.86135	43.85782	34.68301	33.90625

H1–H7 indicates horses’ 1–7, H4 excluded. (B) indicates 168 h baseline. Timepoints in hours are listed as 0, 2, 4, and 6 etc. SD: standard deviation. The inclusion criterion as outlined in Section 2.3 required that horses had data for at least seven out of 10 timepoints to warrant inclusion. Not all horses stood completely quietly for 60 s for all time points.

**Table 2:** Craniocaudal and mediolateral amplitude of displacement analysis.

Cranio-Caudal Displacement			Medio-Lateral Displacement	
	4–12 h	168 h	4–12 h	168 h
**Mean (mm)**	91.229	118.207	38.005	59.904
**Variance**	1444.214	241.203	172.307	55.415
***p*-value**	0.152		0.005	**

CC and ML amplitude of displacement compared between the acutely inflamed period (average of 4–12 h) versus the 168 h post-lameness induction. Significance at *p* = 0.005 denoted by **.

## Data Availability

The displacement data is printed in the paper.

## Supplementary Materials

The following are available online at https://www.mdpi.com/1424-8220/21/4/1286/s1.
