# Supplementary material for: The Feasibility of Equine Field-Based Postural Sway Analysis Using a Single Inertial Sensor
Source: Sensors (Basel). 2021 Feb 11;21(4):1286. doi: 10.3390/s21041286 (PMC7916957; doi:10.3390/s21041286)
Supplement: Supplementary file 1 [file sensors-21-01286-s001.zip › Suppl files/Suppl 1. Postural Sway Stabilograms.pdf]

HORSE 1

Horse 1 Timepoint 0

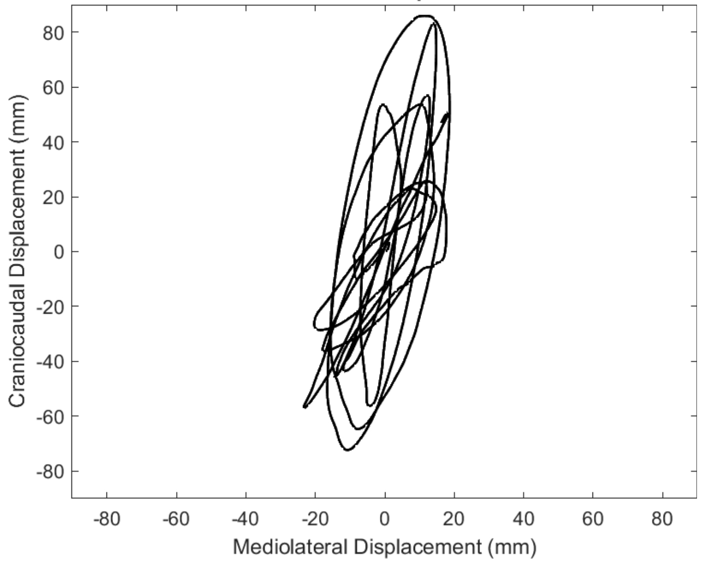

Horse 1 Timepoint 2

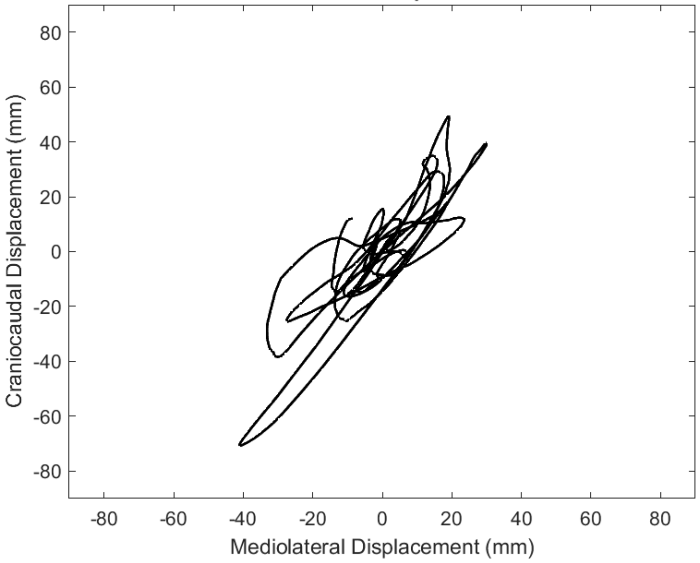

Horse 1 Timepoint 4

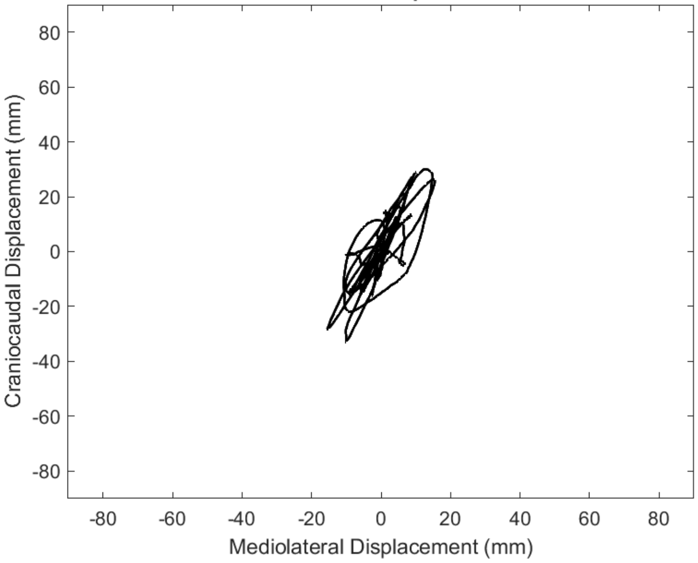

Horse 1 Timepoint 6

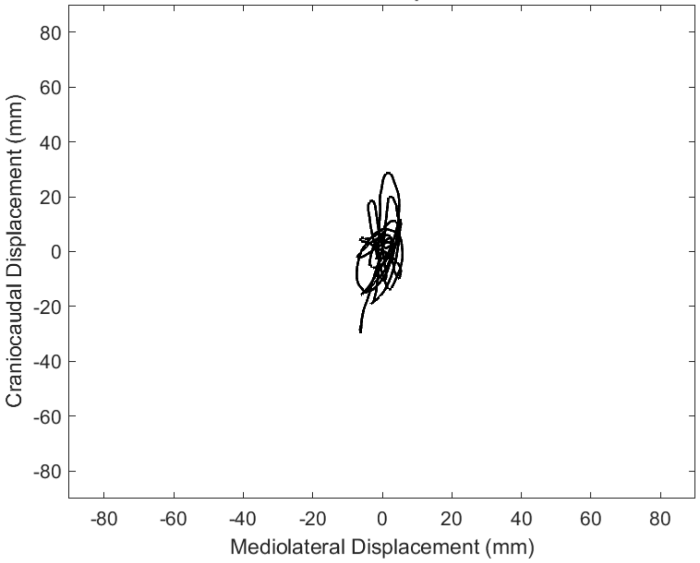

Horse 1 Timepoint 10

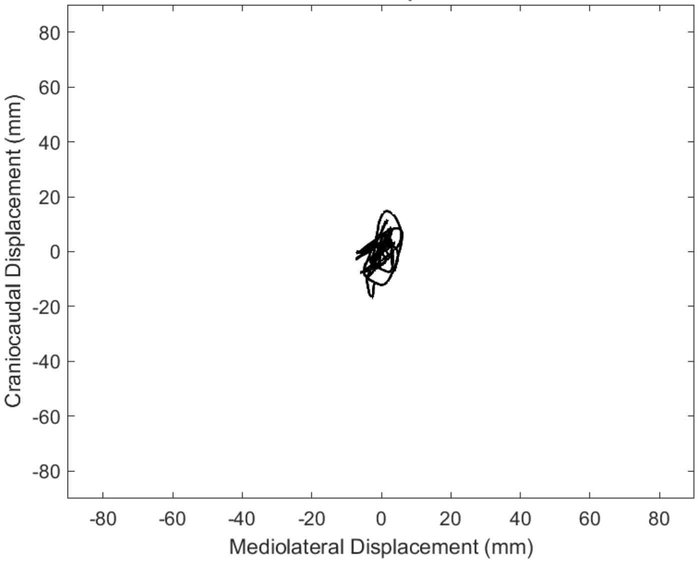

Horse 1 Timepoint 24

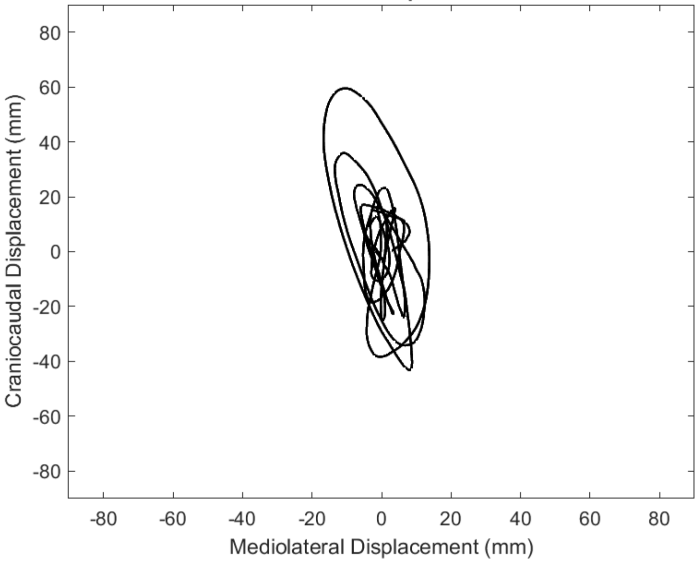

HORSE 2

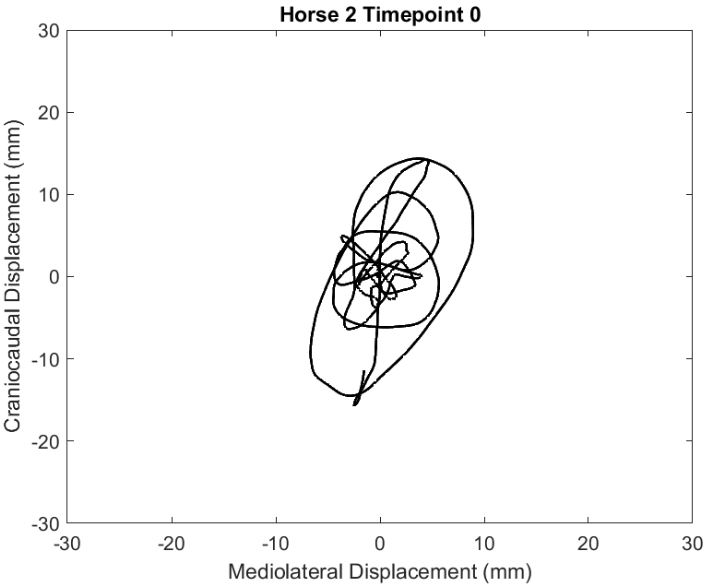
Horse 2 Timepoint 0
Craniocaudal Displacement (mm)
Mediolateral Displacement (mm)
30
20
10
0
-10
-20
-30
-30
-20
-10
0
10
20
30

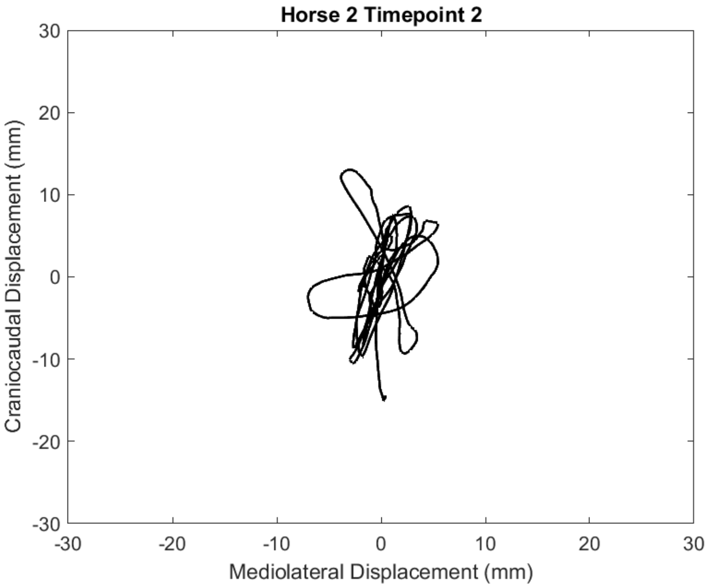
Horse 2 Timepoint 2
Craniocaudal Displacement (mm)
Mediolateral Displacement (mm)
30
20
10
0
-10
-20
-30
-30
-20
-10
0
10
20
30

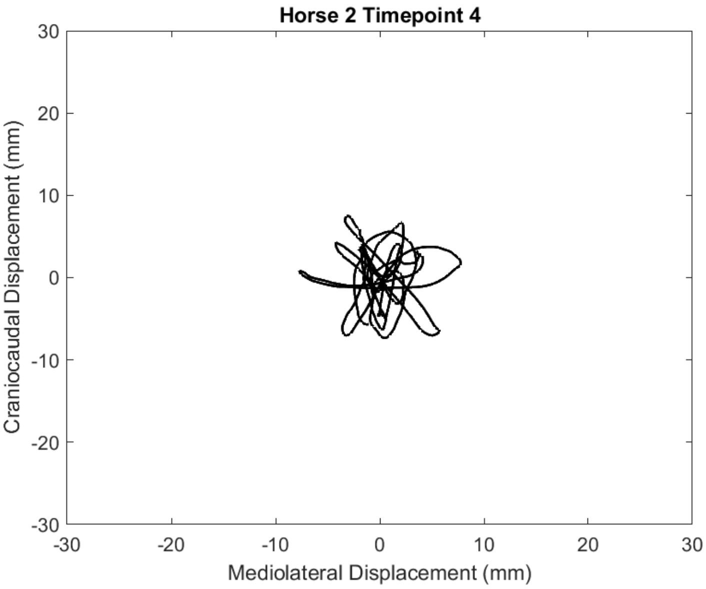
Horse 2 Timepoint 4
Craniocaudal Displacement (mm)
Mediolateral Displacement (mm)
30
20
10
0
-10
-20
-30
-30
-20
-10
0
10
20
30

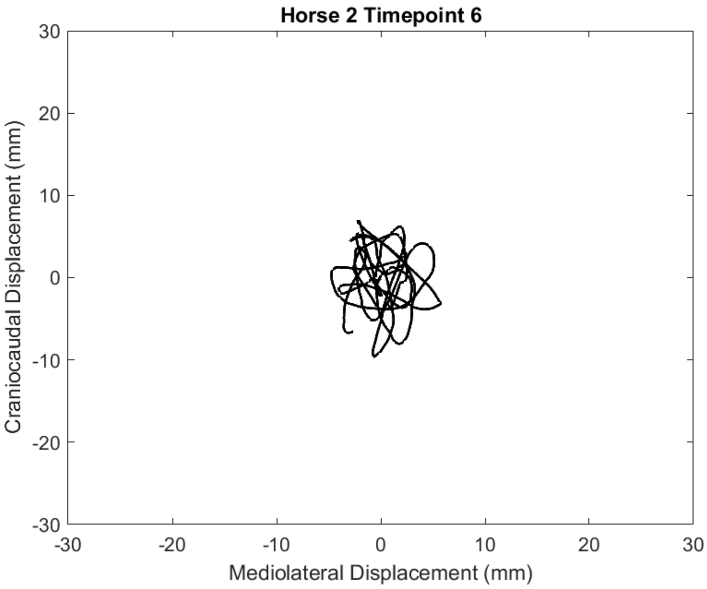
Horse 2 Timepoint 6
Craniocaudal Displacement (mm)
Mediolateral Displacement (mm)
30
20
10
0
-10
-20
-30
-30
-20
-10
0
10
20
30

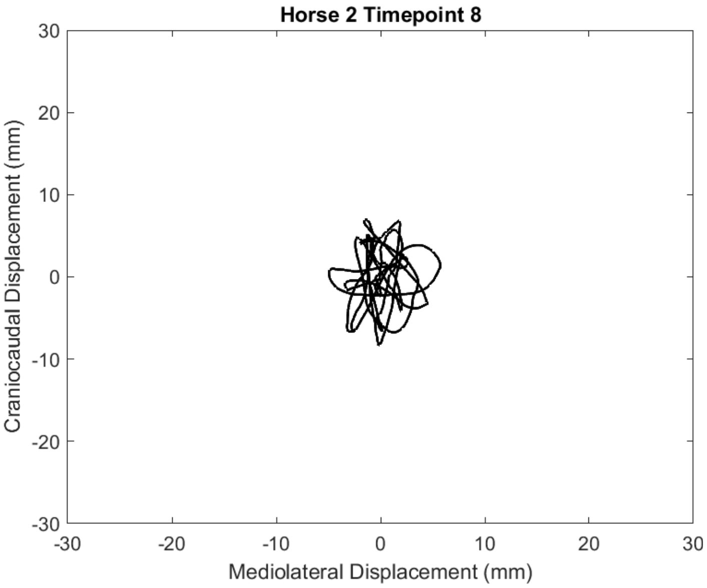
Horse 2 Timepoint 8
Craniocaudal Displacement (mm)
Mediolateral Displacement (mm)
30
20
10
0
-10
-20
-30
-30
-20
-10
0
10
20
30

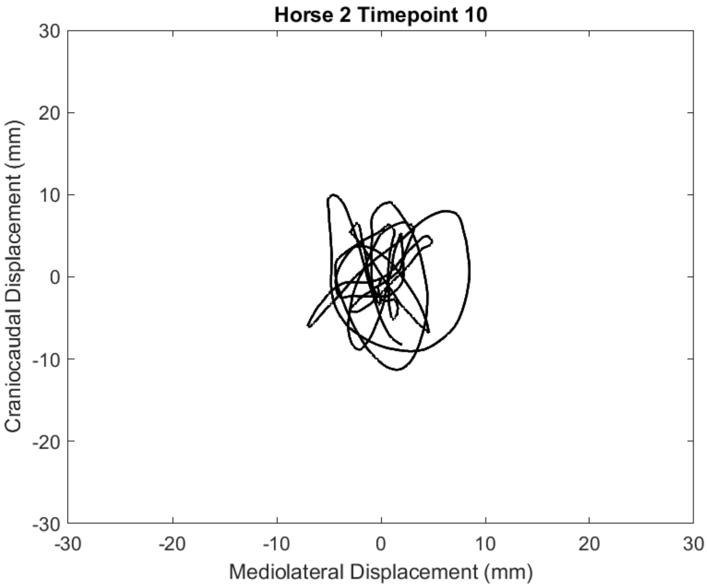
Horse 2 Timepoint 10
Craniocaudal Displacement (mm)
Mediolateral Displacement (mm)
30
20
10
0
-10
-20
-30
-30
-20
-10
0
10
20
30

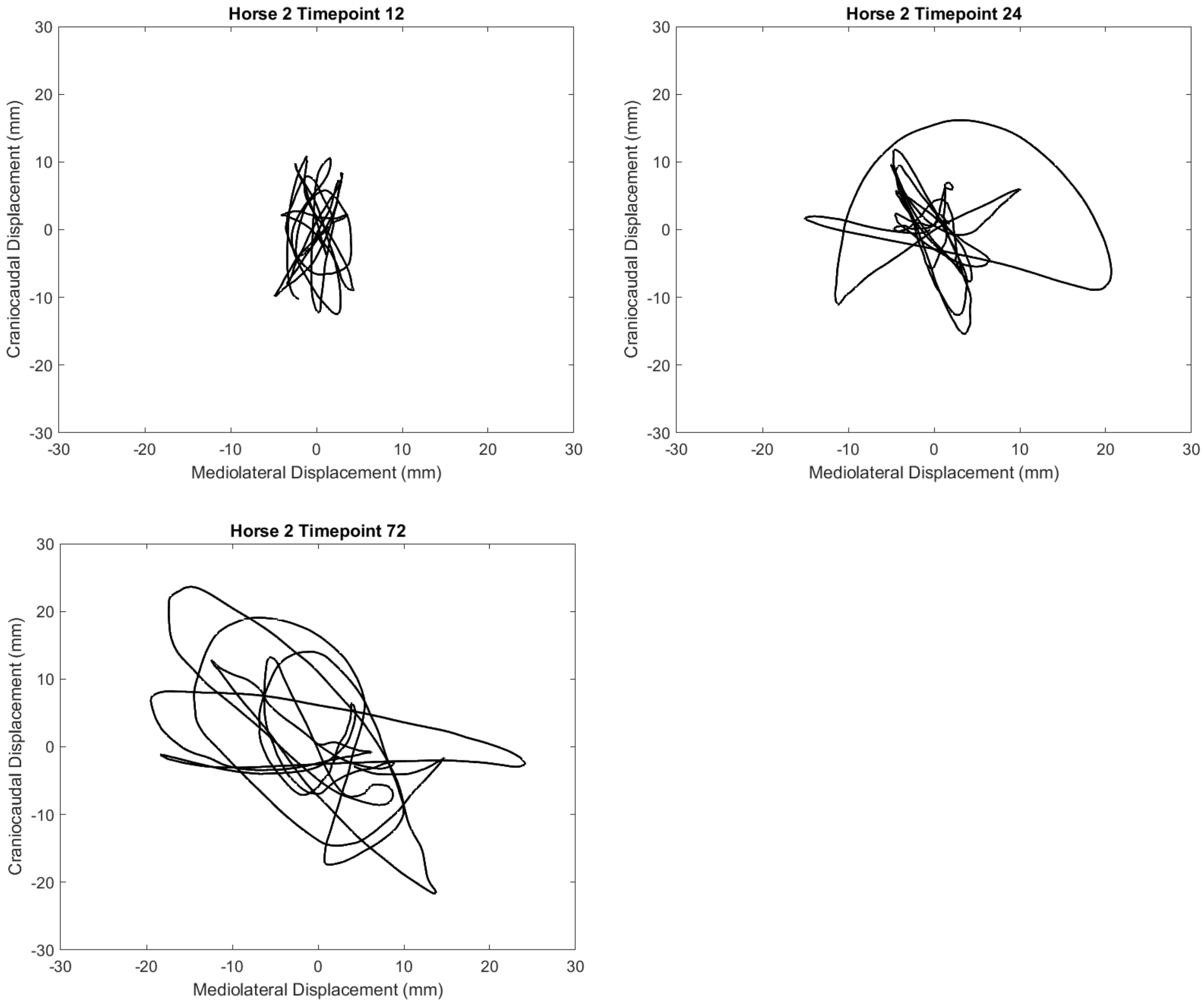
Horse 2 Timepoint 12
Horse 2 Timepoint 24
Horse 2 Timepoint 72
Craniocaudal Displacement (mm)
Mediolateral Displacement (mm)
30
20
10
0
-10
-20
-30

HORSE 3

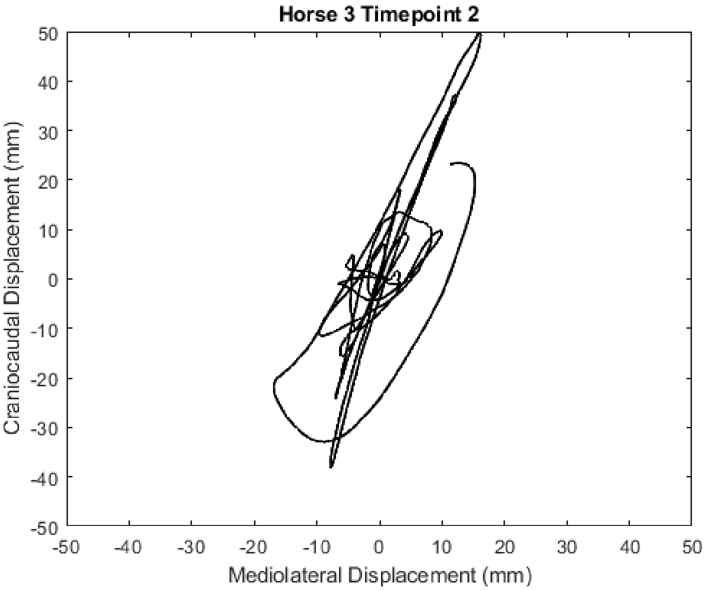
Horse 3 Timepoint 2
Craniocaudal Displacement (mm)
Mediolateral Displacement (mm)

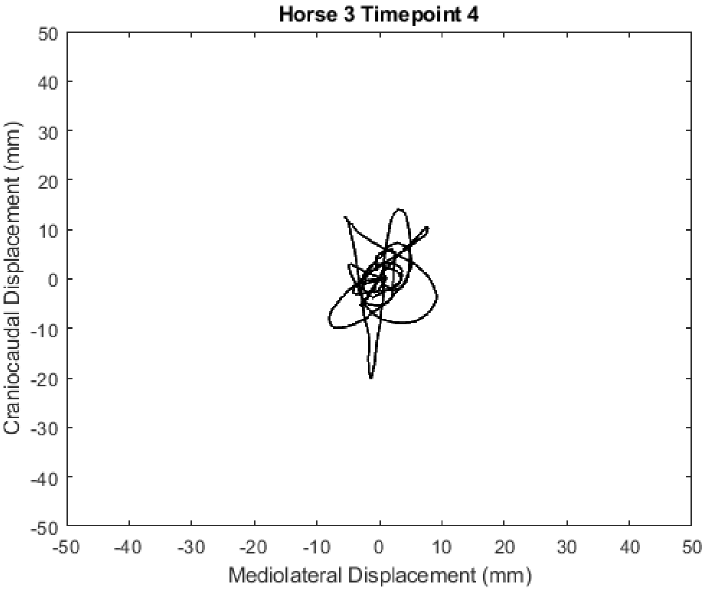
Horse 3 Timepoint 4
Craniocaudal Displacement (mm)
Mediolateral Displacement (mm)

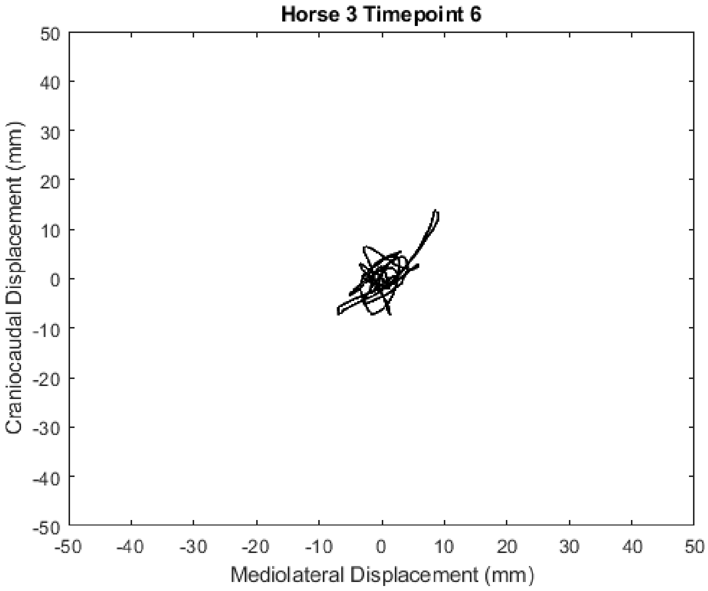
Horse 3 Timepoint 6
Craniocaudal Displacement (mm)
Mediolateral Displacement (mm)

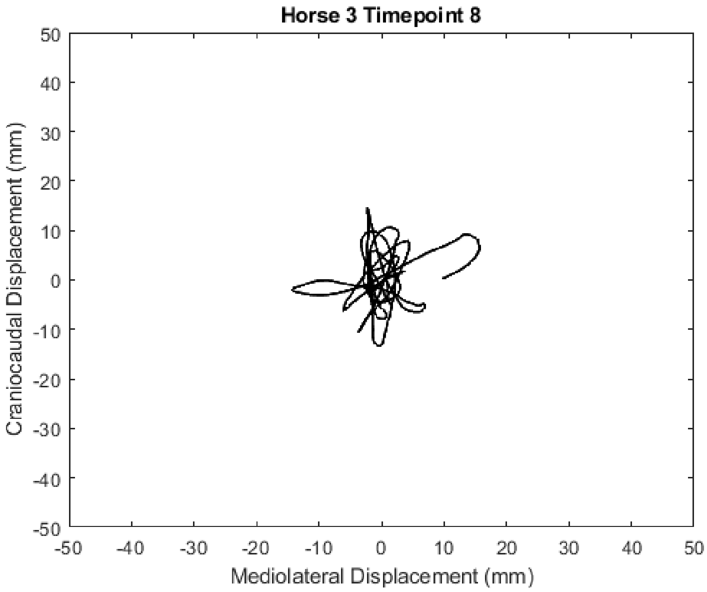
Horse 3 Timepoint 8
Craniocaudal Displacement (mm)
Mediolateral Displacement (mm)

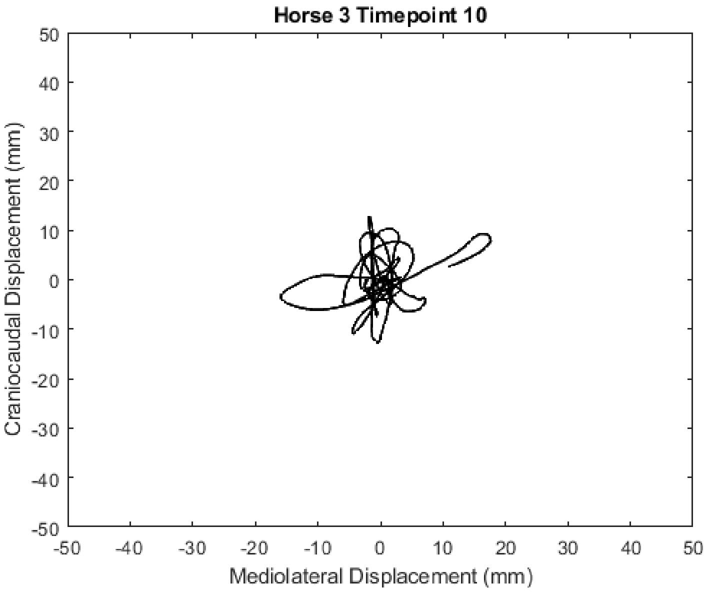
Horse 3 Timepoint 10
Craniocaudal Displacement (mm)
Mediolateral Displacement (mm)

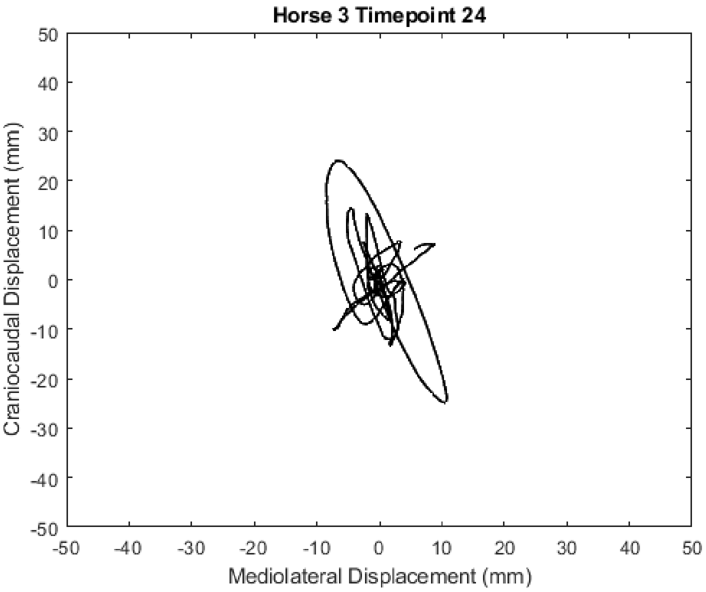
Horse 3 Timepoint 24
Craniocaudal Displacement (mm)
Mediolateral Displacement (mm)

HORSE 5

Horse 5 Timepoint 0

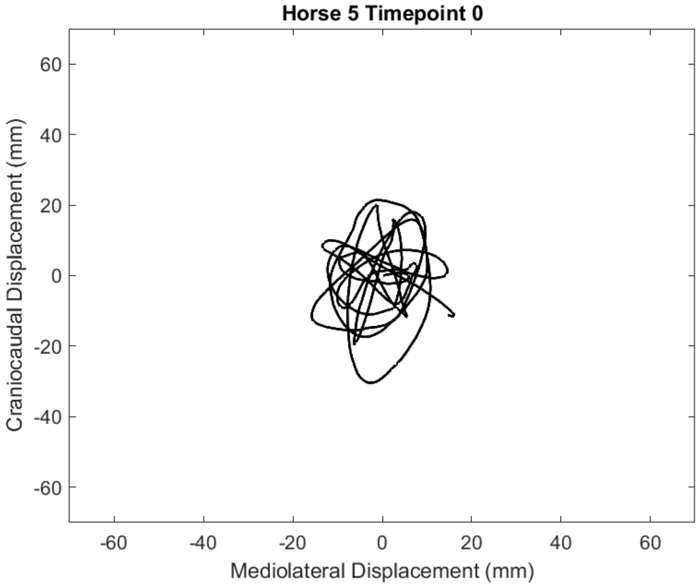

Horse 5 Timepoint 4

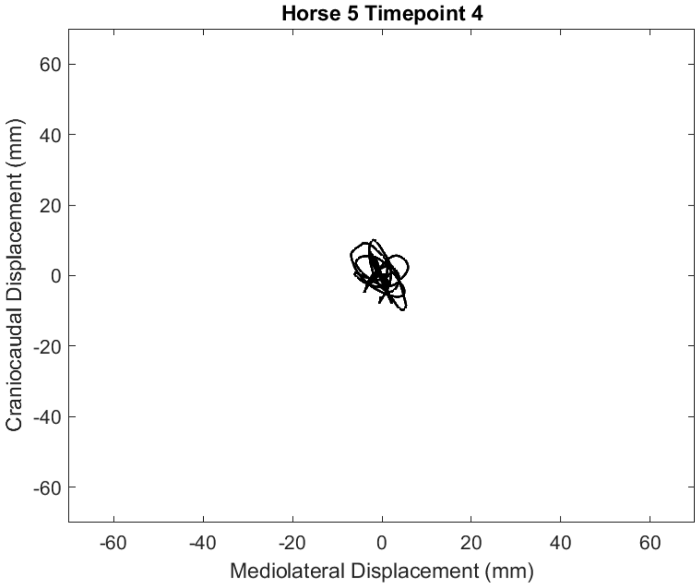

Horse 5 Timepoint 6

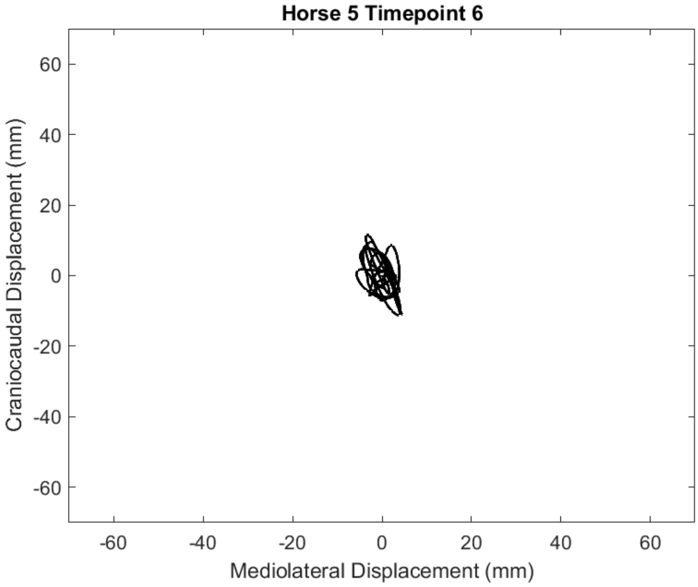

Horse 5 Timepoint 8

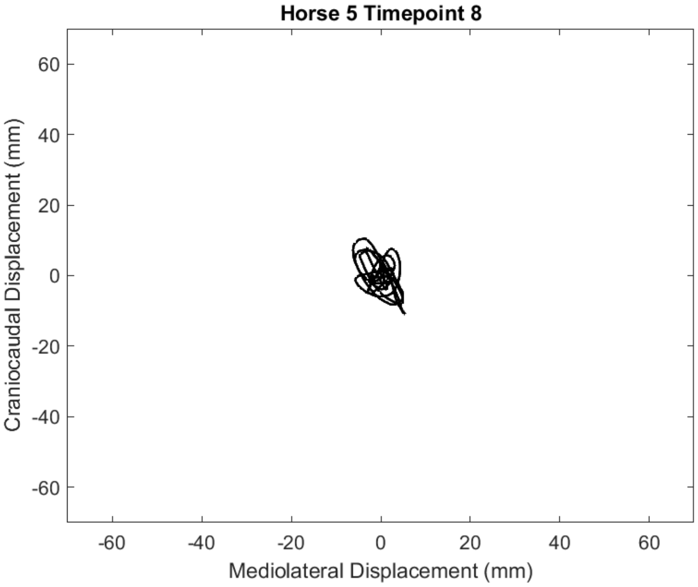

Horse 5 Timepoint 24

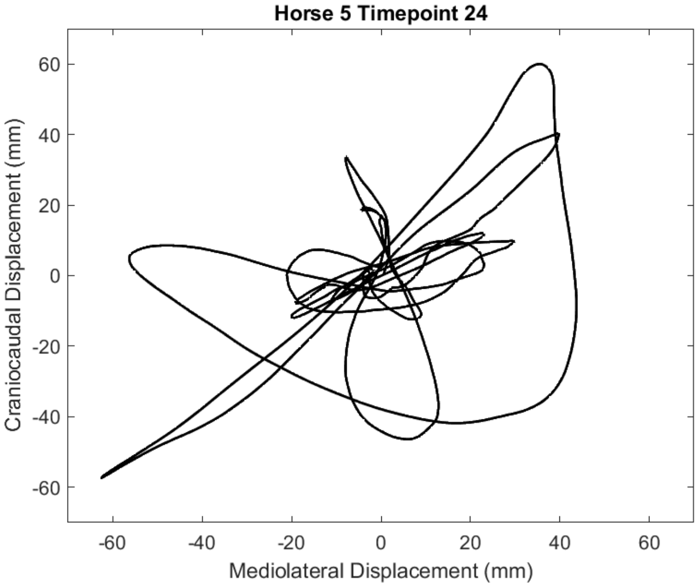

Horse 5 Timepoint 72

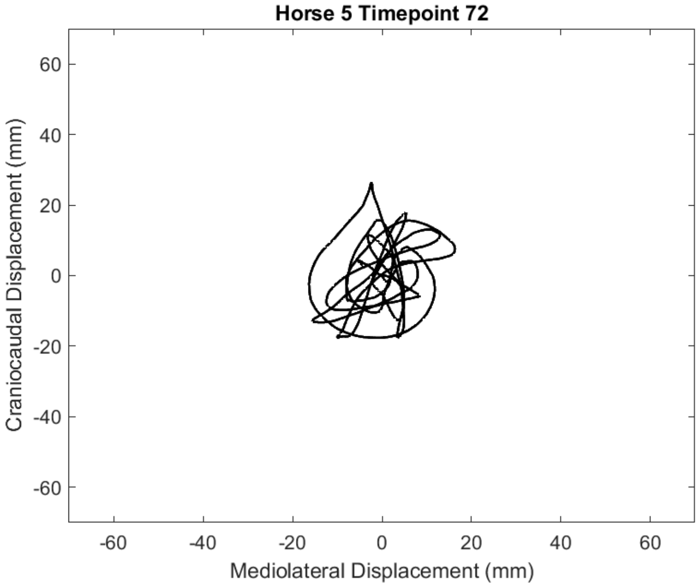

HORSE 6

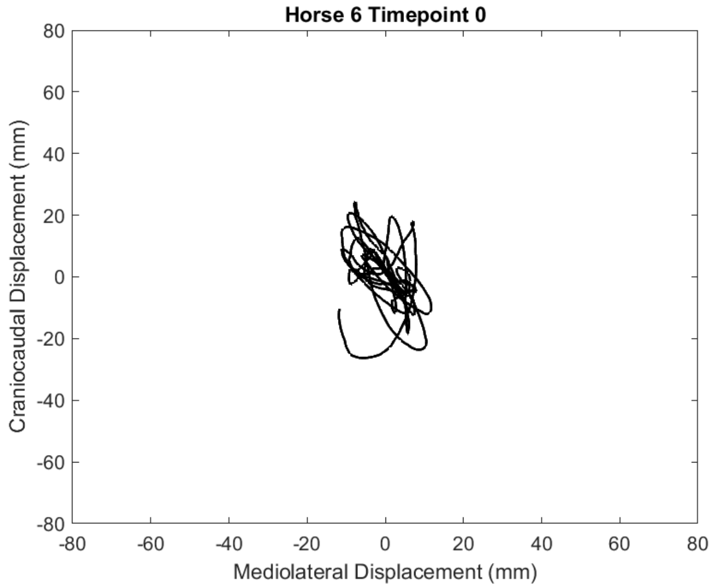
Horse 6 Timepoint 0
Craniocaudal Displacement (mm)
Mediolateral Displacement (mm)

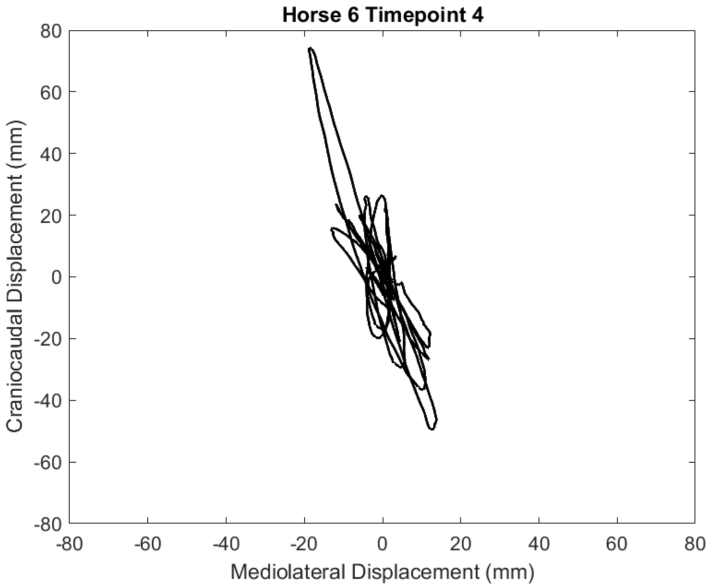
Horse 6 Timepoint 4
Craniocaudal Displacement (mm)
Mediolateral Displacement (mm)

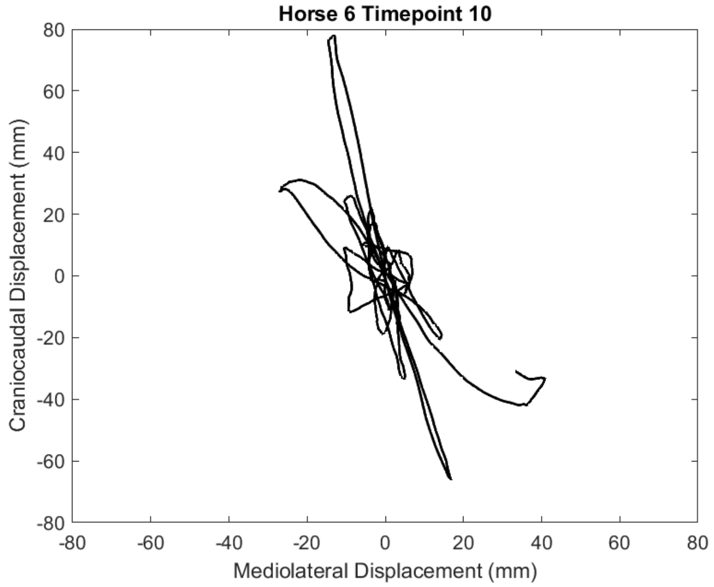
Horse 6 Timepoint 10
Craniocaudal Displacement (mm)
Mediolateral Displacement (mm)

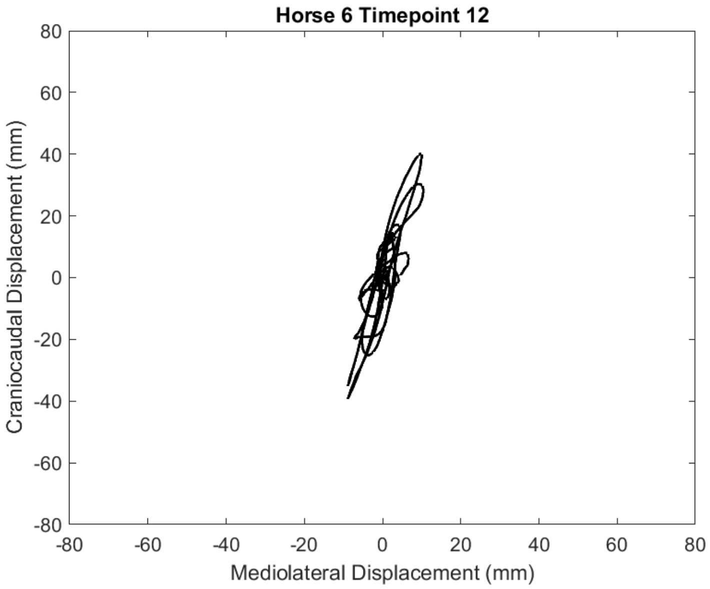
Horse 6 Timepoint 12
Craniocaudal Displacement (mm)
Mediolateral Displacement (mm)

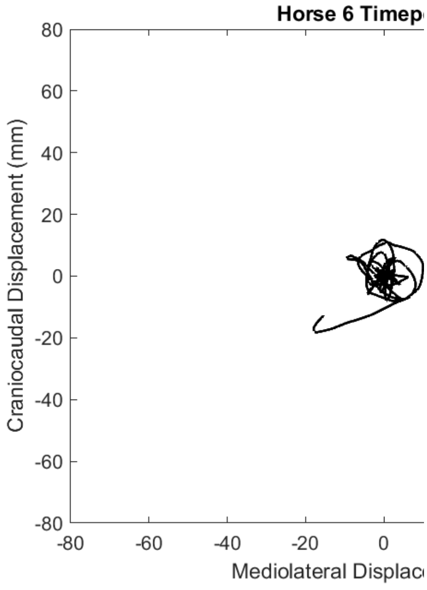
Horse 6
Craniocaudal Displacement (mm)

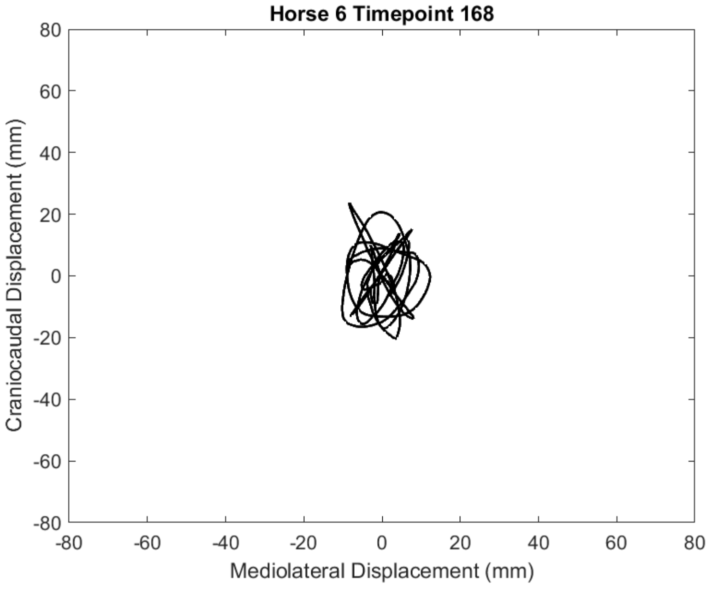
Horse 6 Timepoint 168
Craniocaudal Displacement (mm)
Mediolateral Displacement (mm)

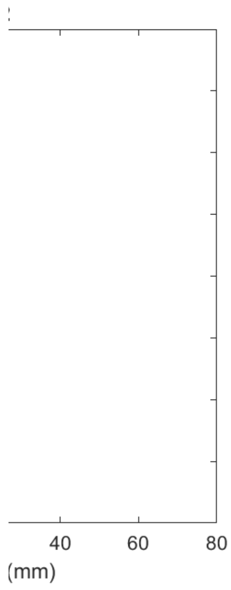
(mm)

HORSE 7

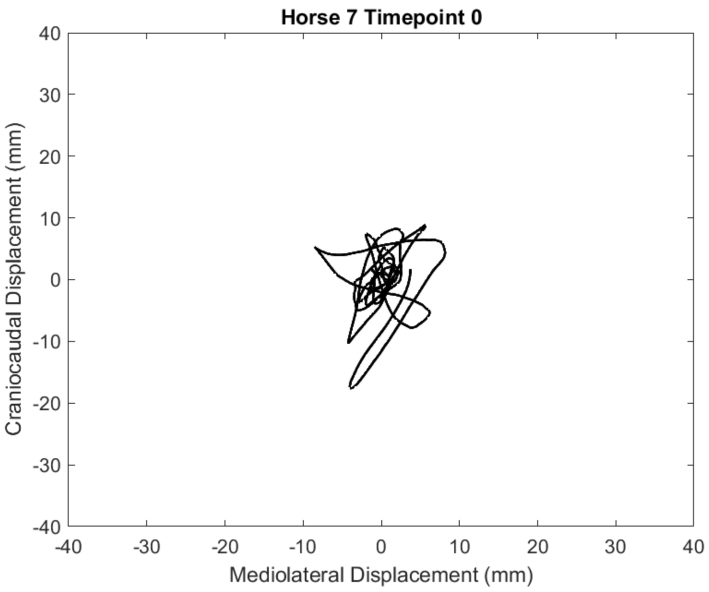
Horse 7 Timepoint 0
Craniocaudal Displacement (mm)
Mediolateral Displacement (mm)

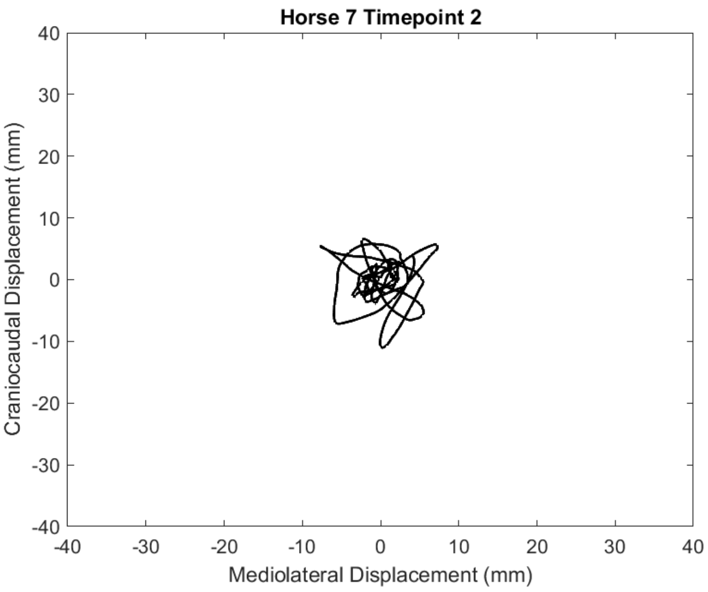
Horse 7 Timepoint 2
Craniocaudal Displacement (mm)
Mediolateral Displacement (mm)

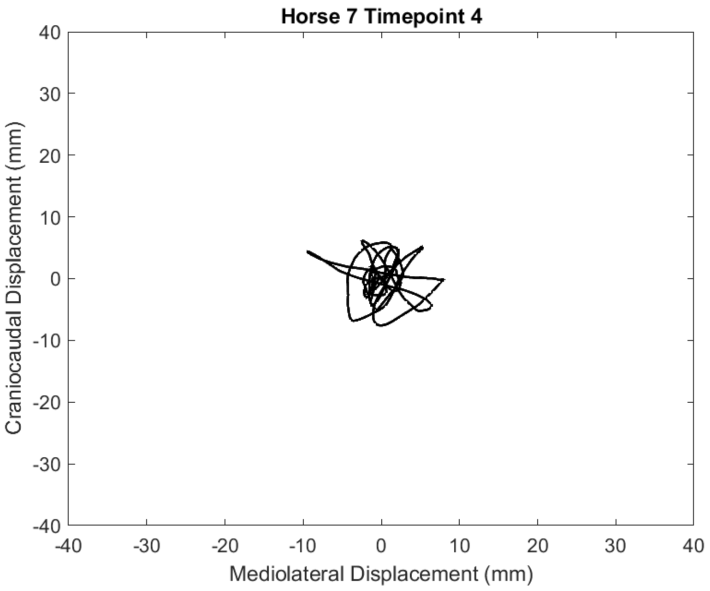
Horse 7 Timepoint 4
Craniocaudal Displacement (mm)
Mediolateral Displacement (mm)

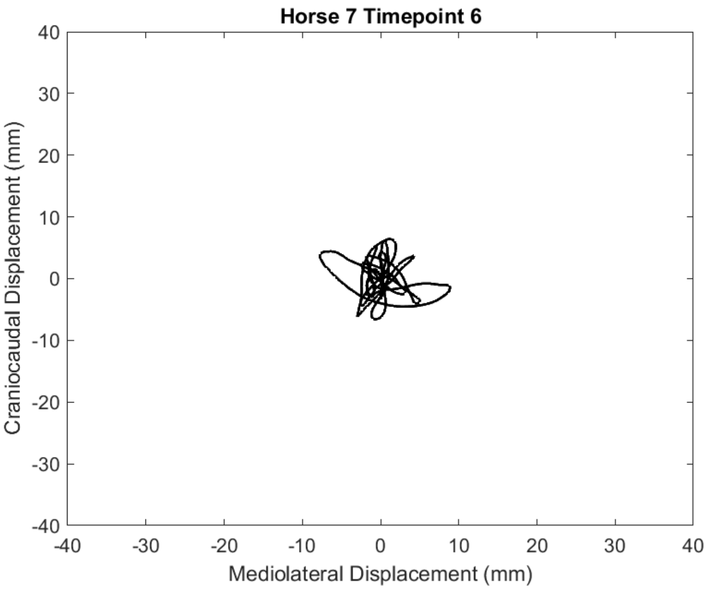
Horse 7 Timepoint 6
Craniocaudal Displacement (mm)
Mediolateral Displacement (mm)

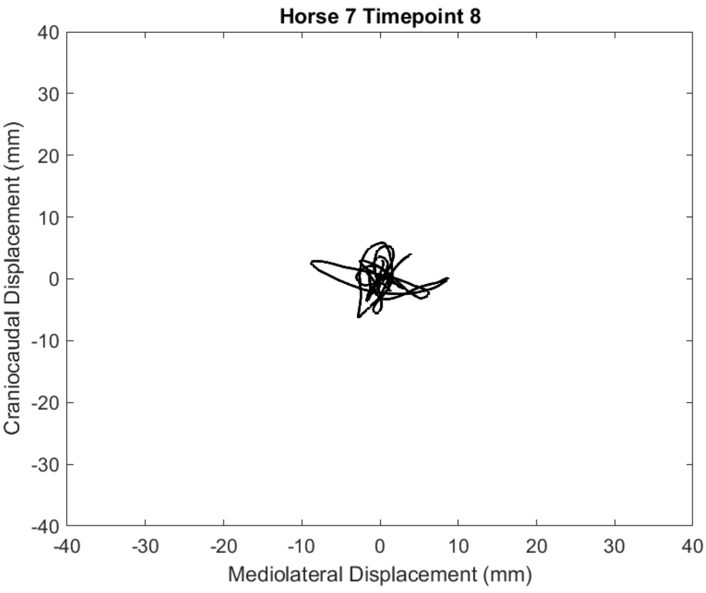
Horse 7 Timepoint 8
Craniocaudal Displacement (mm)
Mediolateral Displacement (mm)

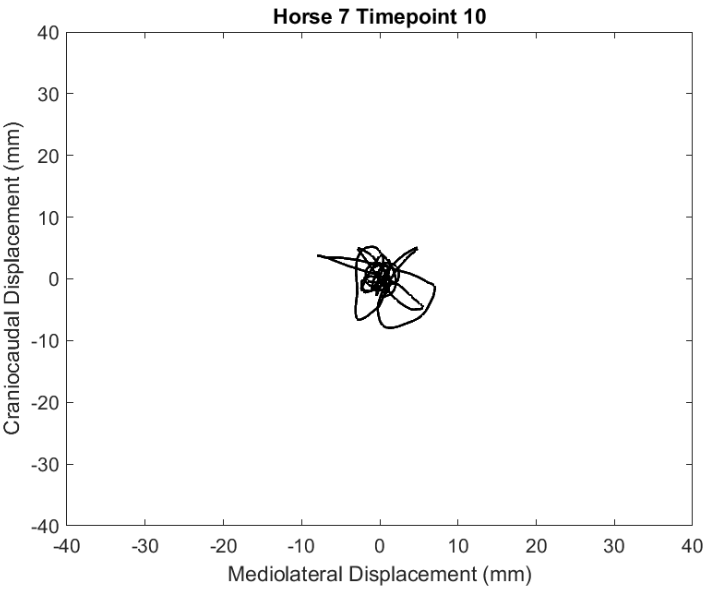
Horse 7 Timepoint 10
Craniocaudal Displacement (mm)
Mediolateral Displacement (mm)

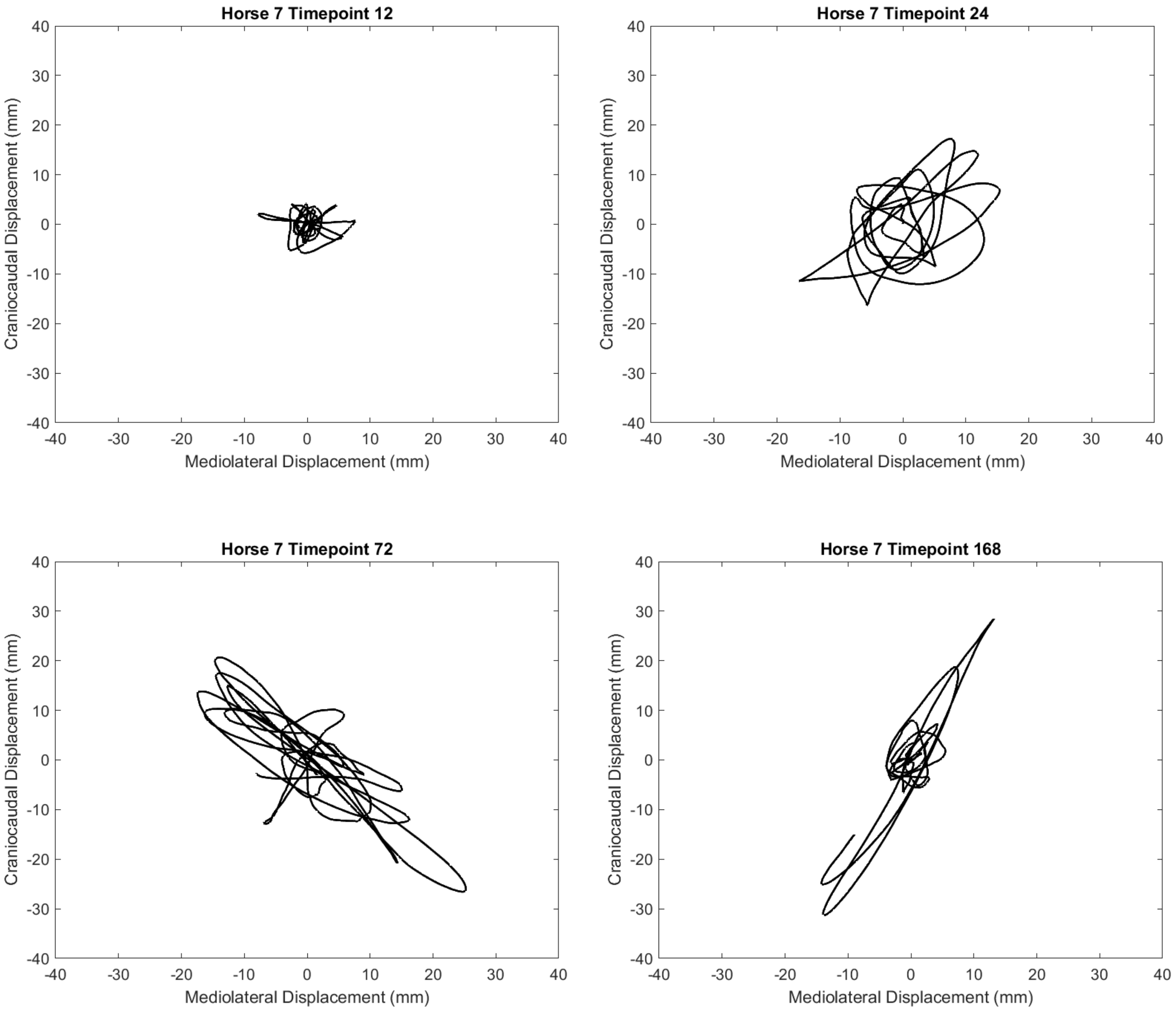
Horse 7 Timepoint 12
Horse 7 Timepoint 24
Horse 7 Timepoint 72
Horse 7 Timepoint 168
Craniocaudal Displacement (mm)
Mediolateral Displacement (mm)
40
30
20
10
0
-10
-20
-30
-40
